# Eclogite resembling metamorphic disequilibrium assemblage formed through fluid-induced metasomatic reactions

**DOI:** 10.1038/s41598-020-76999-y

**Published:** 2020-11-16

**Authors:** Sanghoon Kwon, Vinod O. Samuel, Yungoo Song, Sung Won Kim, Seung-Ik Park, Yirang Jang, M. Santosh

**Affiliations:** 1 Department of Earth System Sciences, Yonsei University, Seoul, 03722 Republic of Korea; 2 Geology Division, Korea Institute of Geoscience and Mineral Resources, Daejeon, 34132 Republic of Korea; 3 Department of Geology, Kyungpook National University, Daegu, 41566 Republic of Korea; 4 Department of Earth and Environmental Sciences, Chonnam National University, Gwangju, 61186 Republic of Korea; 5 School of Earth Sciences and Resources, China University of Geosciences Beijing, 29 Xueyuan Road, Beijing, 100083 China; 6 Department of Earth Sciences, University of Adelaide, Adelaide, SA Australia

**Keywords:** Petrology, Tectonics

## Abstract

Equilibrium omphacite-garnet-bearing mafic rocks have been classified as eclogites, either pristine or retrogressed, that were formed at great depths in the lithosphere. Here we report a unique natural example of eclogite resembling assemblage in disequilibrium formed through fluid-induced metasomatic reactions under the amphibolite to granulite facies. Primarily, the amphibolized protolith experienced a garnet-amphibolite facies metamorphism at ~ 500–700 °C and ~ 0.8–1 GPa. Subsequently, CO_2_ fluid induced fracturing and dissolution-reprecipitation reactions occurred at peak metamorphic conditions of ~ 700 °C and ~ 1 GPa. Occasional omphacite-albite assemblage, which gradually replace diopside-oligoclase symplectite adjacent to albite veins along fractures, indicates fluid-induced coupled dissolution-reprecipitation disequilibrium reactions. Here the albite-omphacite assemblage is in local equilibrium at least on 1 mm length scale, during cooling, below ~ 600 ºC and ~ 1 GPa, within the amphibolite facies conditions. The results from this study clearly suggest that disequilibrium garnet-omphacite assemblage in mafic rocks could be formed by crustal reworking processes below granulite facies conditions, and their textural equilibrium is an important criterion while defining eclogite facies.

## Introduction

Eclogite facies metamorphic rocks formed through subduction and collision processes are common in global orogenic belts^1–12^ (e.g., Usagaran belt^1^, Belomorian Belt^2^, Trans-Hudson orogen^3^, Grenvillian-Caledonian belt^4,5^ Qinling–Dabie–Sulu belt^6^, Appalachian Orogenic Belt^7^, Central Asian Orogenic Belt^8^, Sambagawa belt^9^, Alpine-Himalayan belt^10,11^, Franciscan complex^12^ etc.) created throughout Earth’s history. Equilibrium omphacite-garnet assemblage along with other minor high-pressure phases (e.g., coesite, micro-diamonds) in such rocks are considered as evidence for their burial to mantle depths of about 150 to 200 km, followed by exhumation to crustal levels^13–15^. In exhumed rocks, the relict phases and their trace element characteristics provide robust information on former deep burial of these rocks^16,17^.

Eclogites are metamorphosed mafic rocks that experienced high-pressure metamorphism, composed of garnet and omphacitic clinopyroxene with or without additional minor phases (e.g., phengite, lawsonite, kyanite, coesite, rutile etc.) formed at temperature > 500 °C and pressure > 1.2 GPa^18,19^. The omphacite is a Na-pyroxene with X_Jadeite (Na)_ greater than 0.2 and less than 0.8^20^. This definition of eclogite suggests that garnet and omphacite, as major or relict minerals in rocks, could be an important indicator of previous deep burial of the crustal rocks. Experimental investigations suggest that gabbro/basalt to eclogite transformation is characterized by the formation of garnet-omphacite assemblage, where there is a gradual increase in the pyrope content of the garnet, and plagioclase is absent^21^. However, there are examples of different end members of garnet (Fe, Mg or Mn) equilibrated with omphacite in the presence or absence of plagioclase under high-pressure conditions in metamorphosed sodic trachyte, alkali olivine basalt and eclogitic schists^22,23^. In case of omphacite, experimental studies on the stability of reaction (1) shows that it is stable in a wide range of pressure–temperature conditions^24^. The equilibrium reaction satisfies the eclogite definition (Pressure > 1.2GPa), if X_Jd_ varies from 0.2–0.8 from 500–900 °C^24^.

1 $${\text{NaA1Si}}_{{2}} {\text{O}}_{{6}} + {\text{ SiO}}_{{2}} = {\text{ NaA1Si}}_{{3}} {\text{O}}_{{8}}$$

The above results imply that there exists a large variability in composition of garnet and omphacite, while defining a mafic metamorphic rock as the eclogite. Therefore, in many cases a rock with relict almandine and omphacite (X_Jd_ 0.2–0.3), deprived of any other high-pressure metamorphic evidence, are commonly considered as the retrogressed eclogite^1–3,25,26^, and are considered very important in exploring the geodynamic evolution of the deep crust of our planet^27,28^. However, such commonly assumed equilibrium considerations can lead to misinterpretation of crustal evolution of both subducting and overriding plates during subduction-accretion-collision processes.

For the first time, based on the textural evidence, mineral chemistry, and phase diagram modeling, we have provided an excellent natural example of eclogite resembling disequilibrium garnet-omphacite-bearing mafic metamorphic rock, which might have formed under amphibolite to granulite facies P–T conditions due to fluid-rock interactions. Since Earth’s crust is always exposed to fluid-induced alterations during tectonic events^29,30^, formation of disequilibrium garnet-omphacite-bearing amphibolites in the present cases alert that use of the term eclogite based on garnet and omphacite assemblage alone requires caution to discuss their geodynamic interpretation.

### Amphibolized mafic metamorphic rocks from the central western Korean Peninsula

We have explored metamorphic history of a Neoproterozoic mafic rock exposed along the central western Korean Peninsula of the southwestern Gyeonggi massif in the Hongseong area (Extended data Fig. 1a) as part of the Hongseong-Imjingang belt, also reported as the Gyeonggi marginal belt^31–34^, as a major Phanerozoic orogenic belt of Korea. In this region, multiple generations of Neoproterozoic, Paleozoic and Middle Triassic dismembered mafic–ultramafic rocks intruded into the Paleoproterozoic basement gneisses, and were metamorphosed during Permo-Triassic in age^35–38^. The Neoproterozoic mafic rocks are exposed in the Bibong, Sinri, and Nunggeum areas in Hongseong area^35^ (Extended data Fig. 1b). Mafic rocks in all three locations above have basaltic/gabbroic composition^35^, and are largely amphibolized with minor garnet and clinopyroxene bearing local pockets/domains^37^. Presence of omphacite was reported only from the mafic rocks in the Bibong area^26,36,37,39^. Thus, in this study, we have focused mainly on the metamorphosed mafic rocks in the Bibong area of the Hongseong-Imjingang belt (Extended data Fig. 1b).

Previous zircon U–Pb geochronology on this mafic rock yielded Neoproterozoic protolith age of *ca.* 803 ± 24 Ma, and middle to upper Triassic metamorphic age of *ca.* 240 to 230 Ma^35^. The Sm–Nd whole rock-garnet internal isochron ages from this rock are 258 ± 11 Ma and 225 ± 6.6 Ma^36^. The previous geochemical studies using bulk major, trace and REE data shows that the magma source of this rocks is E-MORB-type fertile asthenospheric mantle affected by crustal contamination in a forearc setting during Neoproterozoic subduction event^35^. Subsequently in Permo-Triassic, high-grade metamorphism led to the formation of garnet, omphacite, clinopyroxene assemblage in this rock^26,36,37,39^.

The Bibong outcrops are enclosed within the amphibolite facies Paleoproterozoic and Middle Paleozoic felsic gneisses without any obvious boundary or shear zones^36^ (Extended data Fig. 1b). Except this small body, no other eclogite were found in the Korean Peninsula, and the various amphibolite facies meta-igneous rocks including serpentinized ultramafic rocks, metavolcanics, metasyenite, amphibolite, and amphibole schist also occur as isolated bodies within the basement gneisses^35^. This difference in metamorphic grade with surrounding amphibolite faces basement gneisses were an enigma in interpreting the geology of the western Korean Peninsula. Our field observations show that the lenticular/oval shaped outcrop with a size of 500 m × 200 m is mostly amphibolized (> 90%), and made up of the dark greenish amphiboles. Within the amphibolized outcrop (Extended data Fig. 2a) there are local high-grade metamorphic domains/pockets bearing feldspar, garnet and clinopyroxene (< 10%). White feldspar-rich veins occur along the fractures, ranging in width from mm to a few cm in thickness (Extended data Fig. 2b–d). We collected samples of the mafic rock with and without garnet, clinopyroxene and feldspar for detailed microstructural study.

### Details of microstructural observations

Detailed microstructural relations were studied using three representative thin-sections from the amphibole-enriched part (TS1), domain enriched in amphibole, garnet, diopside and plagioclase (TS2), and omphacite-bearing domain (TS3).

**Figure 1:**
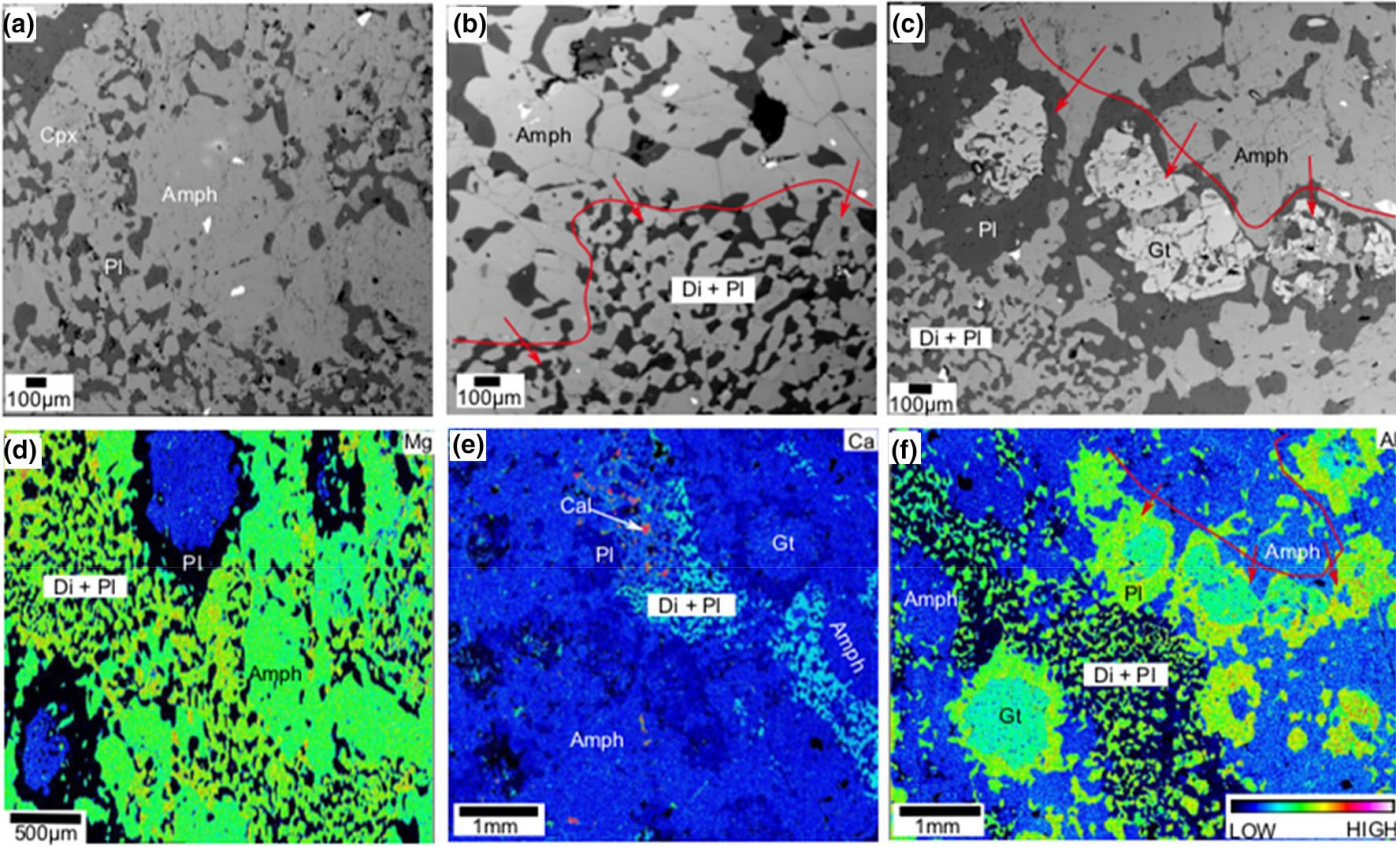
Back-Scattered Electron (BSE) images and elemental maps showing textures of amphibole reaction rims in local domains within TS2 and TS3 thin-sections. (**a**) Diopside-plagioclase symplectites on the amphibole grain boundaries. (**b**) Red boundary separates amphibole from symplectite. Red arrows show direction of symplectite formation from amphibole. (**c**) Garnet grains along with plagioclase formed by amphibole breakdown. Red boundary separates amphibole from symplectite. Red arrows show direction of symplectite formation from amphibole. (**d**) Mg composition map showing amphibole reaction rims with high-Mg content in diopside. (**e**) Ca composition map shows amphibole breakdown textures, here calcite grains appears red due to high Ca content. (**f**) Al composition map shows amphibole breakdown textures, here plagioclase appears green due to high Al content. Di-diopside; Gt-garnet; Pl-Plagioclase (Andesine–Oligoclase); Amph-Amphibole; Low and High represents color with respect to concentration of elements.

The TS1 is densely packed with dark greenish amphibole laths ranging in size from ~ 2 to 5 mm. The grain boundaries are sharp without any reaction textures. Occasionally, discrete minor plagioclase, biotite and ilmenite are present in the matrix. The TS2 and TS3 samples consist mostly of similar ~ 2 to 5 mm size laths of dark greenish to brownish amphiboles. Compositionally they are magnesio-hornblende to magnesio-ferri-hornblende in TS2 and pargasite to ferro-pargasite in TS3^40^ (Extended data Table S1). Locally, these are intensely fractured and are breaking down to form symplectitic textures along grain boundaries (Fig. 1). The main minerals observed in the symplectites are diopside (Extended data Table S2), garnet (56–57% almandine, 25–27% grossular and only 15–16% pyrope content; Extended data Table S3), and plagioclase that has andesine–oligoclase composition (Extended data Table S4; Fig. 1a–f). In back-scattered electron (BSE) image, the diopside shows oval-shaped distinct grains or interconnected grains (0.2 to 0.5 mm) with clear polyhedral boundaries (Fig. 1a,b). The mineral is pristine, undeformed and unfractured without any later overprinting of any other minerals around or within them (Fig. 1a–c). In another area, garnet grains along with plagioclase are formed from amphiboles (Fig. 1c). Compared to diopside, rounded to sub-rounded garnet grains are large in size (1 to 2 mm). Such garnet-plagioclase assemblage transforms to diopside-plagioclase symplectite further away from amphiboles (Fig. 1c). Fully grown garnets have fractures filled with plagioclase (Fig. 1c). Occasionally diopside/amphibole is present within garnet (details of the textures are given in following sections). Compositional maps of Mg, Ca and Al clearly show textures, representing breaking down of large amphibole laths to symplectites along their boundaries (Fig. 1d–f). The microstructural relationships in these samples suggest that amphiboles are breaking down to form garnet-plagioclase, diopside-plagioclase symplectites.

**Figure 2:**
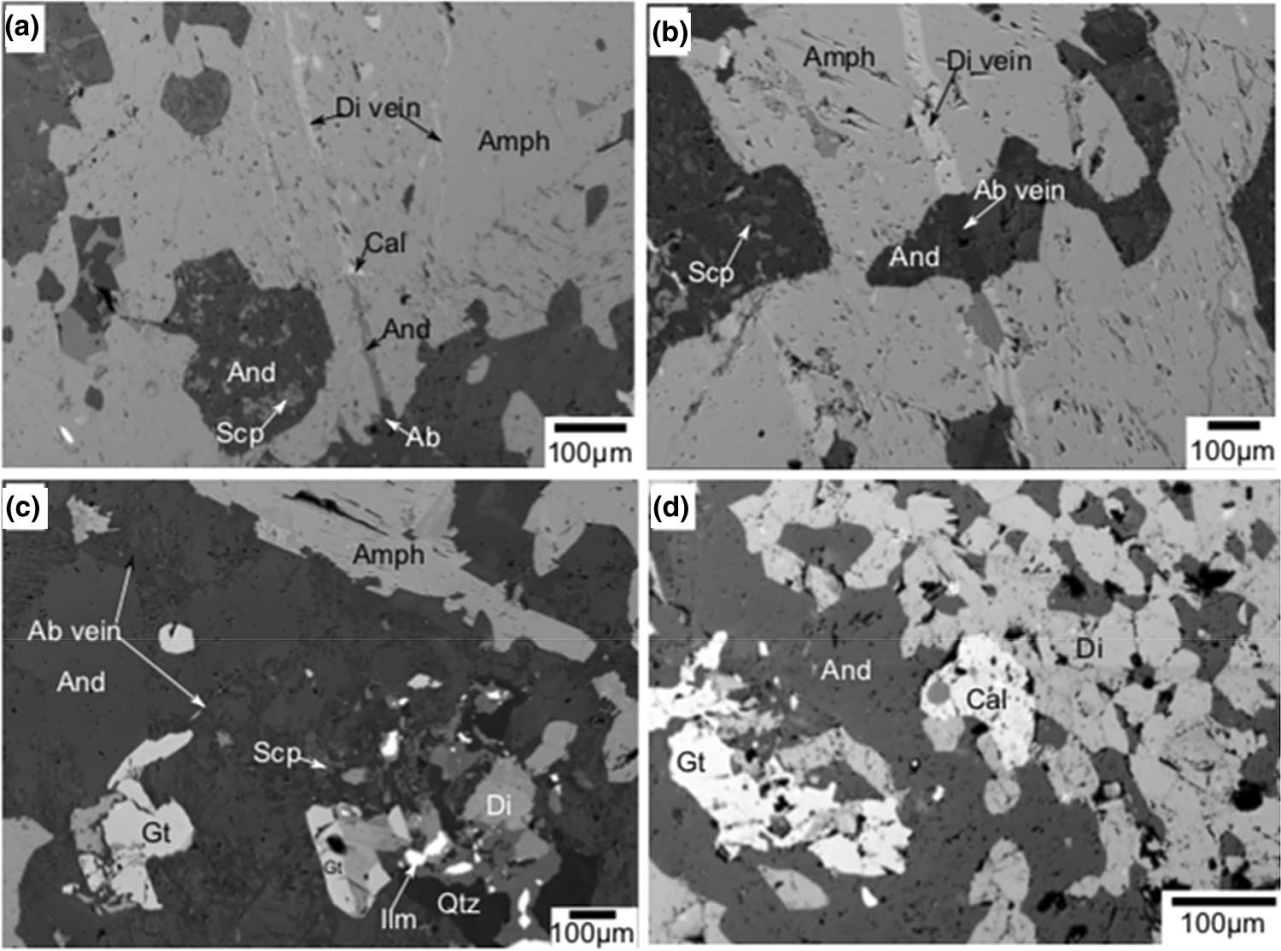
Back-Scattered Electron (BSE) images showing microstructures in TS2 and TS3 samples. (**a**) Minerals such as albite (dark), andesine (grey), diopside (bright) and white calcite precipitate as veins in fractures. (**b**) Albite (dark), andesine (grey), diopside (bright) precipitate as veins in fractures that cross-cut the amphibole boundaries. (**c**) Albite networks forms along grain boundaries of the andesine along with garnet, diopside, scapolite and ilmenite around amphibole boundary. (**d**) A large calcite grains are precipitated near garnet, plagioclase, diopside symplectite region. Di-diopside; Gt-garnet; And-andesine; Ab-albite; Amph-Amphibole; Scp-scapolite; Cal-calcite.

Further microstructural observations show that albite (dark; Extended data Table S5), andesine–oligoclase (grey), diopside (bright) and white calcite are precipitated as veins along fractures in amphibole laths (Fig. 2a). Diopside is precipitated as vein within fracture inside amphibole and albite is precipitated as vein when the fracture extends through andesine–oligoclase (Fig. 2b). Thin albite networks formed along grain boundaries of the andesine–oligoclase matrix outside amphibole laths (Fig. 2c). Garnet, diopside, scapolite (meionite-Extended data Table S6) and ilmenite are also formed in the same areas (Fig. 2c,d), and large calcite grains (Extended data Table S7) are precipitated near garnet, plagioclase, diopside symplectite region (Figs. 1e, 2d).

**Figure 3:**
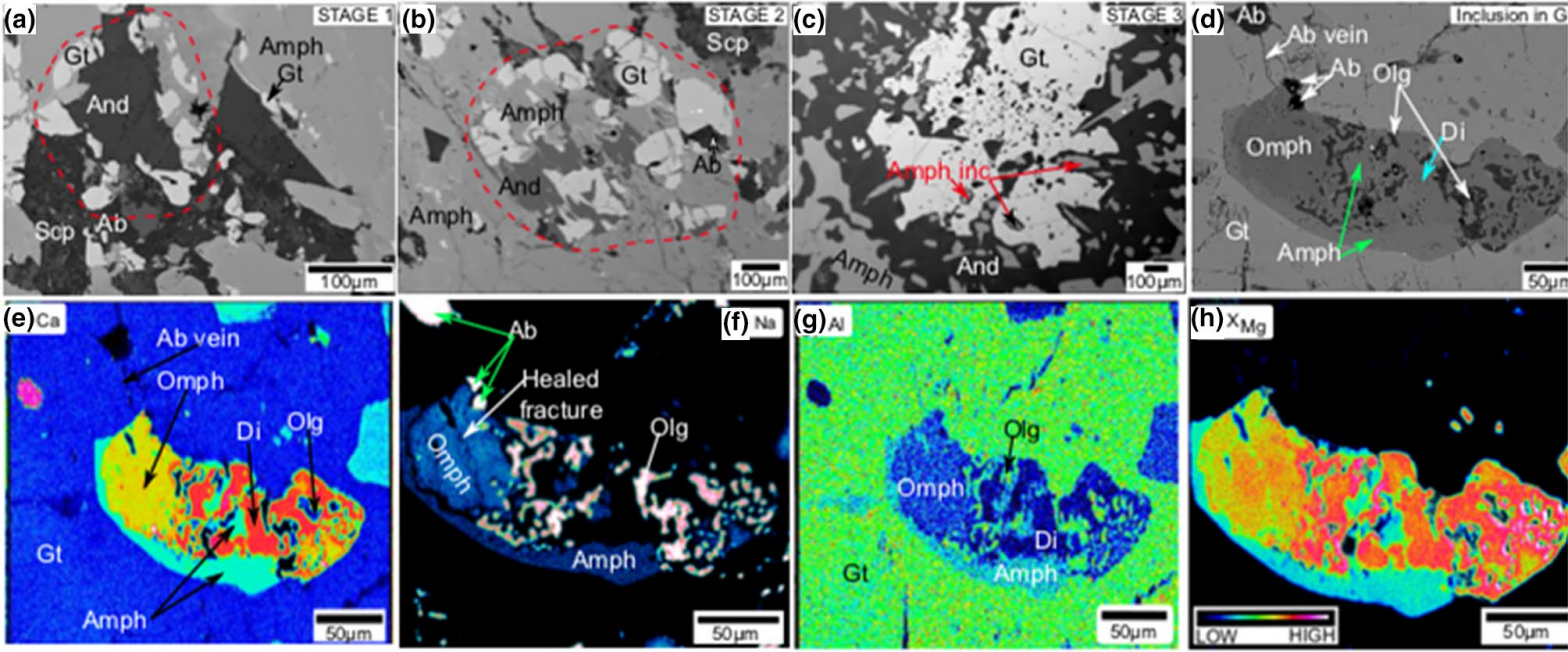
Different stages of garnet formation in the different domains of TS2 and TS3 thin-sections. (**a**) Anhedral garnet crystals formed along the amphibole grain boundaries. (**b**) Larger garnet grains along with andesine on amphibole boundaries. (**c**) Relict amphibole grain is getting enclosed by growing garnets. (**d**) Breakdown of amphibole inclusions within garnet. (**e**–**h**) Elemental compositional maps show more details of breaking down of amphibole inclusions in garnet. Compositional variations in Ca (**e**), Na (**f**), Al (**g**) and X_Mg_ (**h**) are shown in the maps. High-Ca, low-Al represents diopside. Albite can be identified by high Na, high Al and absence of Mg. Omphacite has high-Ca, high-Na, low-Al, high-Mg compared to those of amphibole. Di-diopside; Gt-garnet; And-andesine; Olg-oligoclase; Ab-albite; Amph-Amphibole; Omph-omphacite; Scp-scapolite.

Different stages of garnet formation, with respect to grain size, are identified in different domains of the TS3 and TS2 thin-sections (Fig. 3a–d). Initial stage is represented by small anhedral garnet crystals formed along the amphibole grain boundaries (Fig. 3a). Larger garnet grains near the amphibole boundaries could be considered as representing the second stage of garnet growth (Fig. 3b). Stage 3 is represented by relict amphibole grains enclosed by growing garnet grains (Fig. 3c). This accounts for the occurrence of amphibole inclusions in the garnet grains during their growth. The final stage is represented by the breakdown of amphibole inclusions within garnet (Fig. 3d). Here, near to albite veins across garnet, amphibole breakdowns to symplectites. Diopside-oligoclase and omphacite (Extended data Table S8)-albite symplectites might have formed during decomposition of amphibole inclusions in garnet.

Elemental compositional maps show more details of breaking down of amphibole inclusions in garnet (Fig. 3e–h). Compositional variations in Ca (Fig. 3e), Na (Fig. 3f), Al (Fig. 3g) and X_Mg_ (Fig. 3h) are shown in the maps. Here the albite vein and albite grains have direct contact with omphacite (Fig. 3d,f). A healed fracture running through center of the omphacite grain is shown in Fig. 3f. Relicts of previous amphibole inclusions are still present in this domain. The diopside-oligoclase symplectite has comparatively larger grain size than the omphacite-albite assemblage. The omphacite-albite assemblage has a sieve or mesh-like texture, where omphacite appears like a network with dispersed albite grains. They occur adjacent to albite-bearing vein in the fracture within garnet.

**Figure 4:**
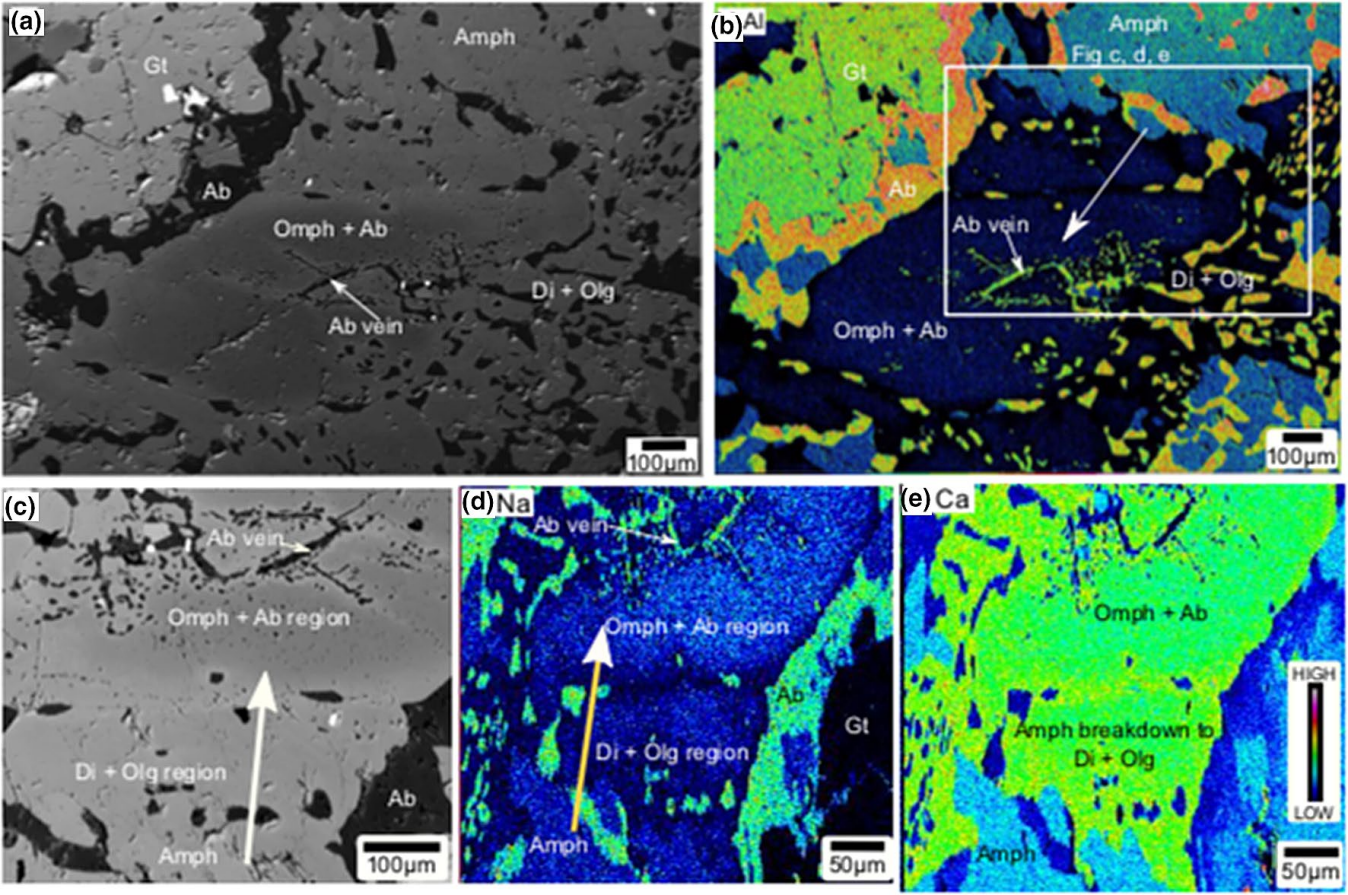
(**a**,**c**) Amphibole breaking down to symplectites of diopside-oligoclase and omphacite-albite. Diopside-oligoclase near the amphibole grain boundary transforms to omphacite-albite symplectite near the albite in vein-filled fracture. In this alteration zone the area with omphacite-albite symplectite appears darker in a BSE image due to finer albite grains. (**b**) Al map shows low-Al content in the pyroxene, moderately high Al content in the amphibole compared diopside, high-Al content in garnet, very high-Al content in oligoclase and albite. (**d**) Na map clearly shows the sieve texture of albite and omphacite. (**e**) Ca map shows high Ca content in amphibole compared to pyroxenes and plagioclase. Diopside has a relatively higher content in Ca compared to omphacite. Di-diopside; Gt-garnet; Olg-oligoclase; Ab-albite; Amph-Amphibole; Omph-omphacite.

Symplectites of diopside-oligoclase and omphacite-albite also occur outside the garnet grains (Fig. 4). Here, omphacite appears to be forming in a pervasive manner around albite-bearing vein (Fig. 4a). Al compositional map shows that omphacite formation occurs where amphibole breaks down to diopside-oligoclase symplectite near the amphibole grain boundary (Fig. 4b). Omphacite-albite symplectite is present further away from the amphibole, adjacent to albite precipitated in vein-filled fractures. Similar to the textures observed in garnet, the omphacite-albite symplectite shows sieve or mesh texture consisting of omphacite and a network of dispersed minute albite grains (Fig. 4c). In this alteration zone, the area with omphacite-albite symplectite appears darker in the BSE image due to finer albite grains (Fig. 4c). The albite phases in the albite-omphacite symplectite become larger near albite veins. The sieve texture of albite and omphacite is obvious in the Na, Ca, Al maps (Fig. 4b–d).

Our detailed petrographic studies show that amphibole, plagioclase (andesine–albite), garnet, clinopyroxene (diopside–omphacite), scapolite (meionite) and calcite are the main mineral assemblage in the metamorphosed mafic rocks from this study. Textural relations show that amphibole laths have broken down to form garnet-(andesine-oligoclase), diopside-(andesine-oligoclase), omphacite-albite assemblages along with scapolite and calcite in the localized areas and within garnets. Sub-micron scale disequilibrium omphacite-albite symplectite forms rarely near thick albite bearing veins. Please see “Extended text 1” for the detailed mineral chemistry description.

### Phase diagram modeling and pressure (P)–temperature (T) path

**Figure 5:**
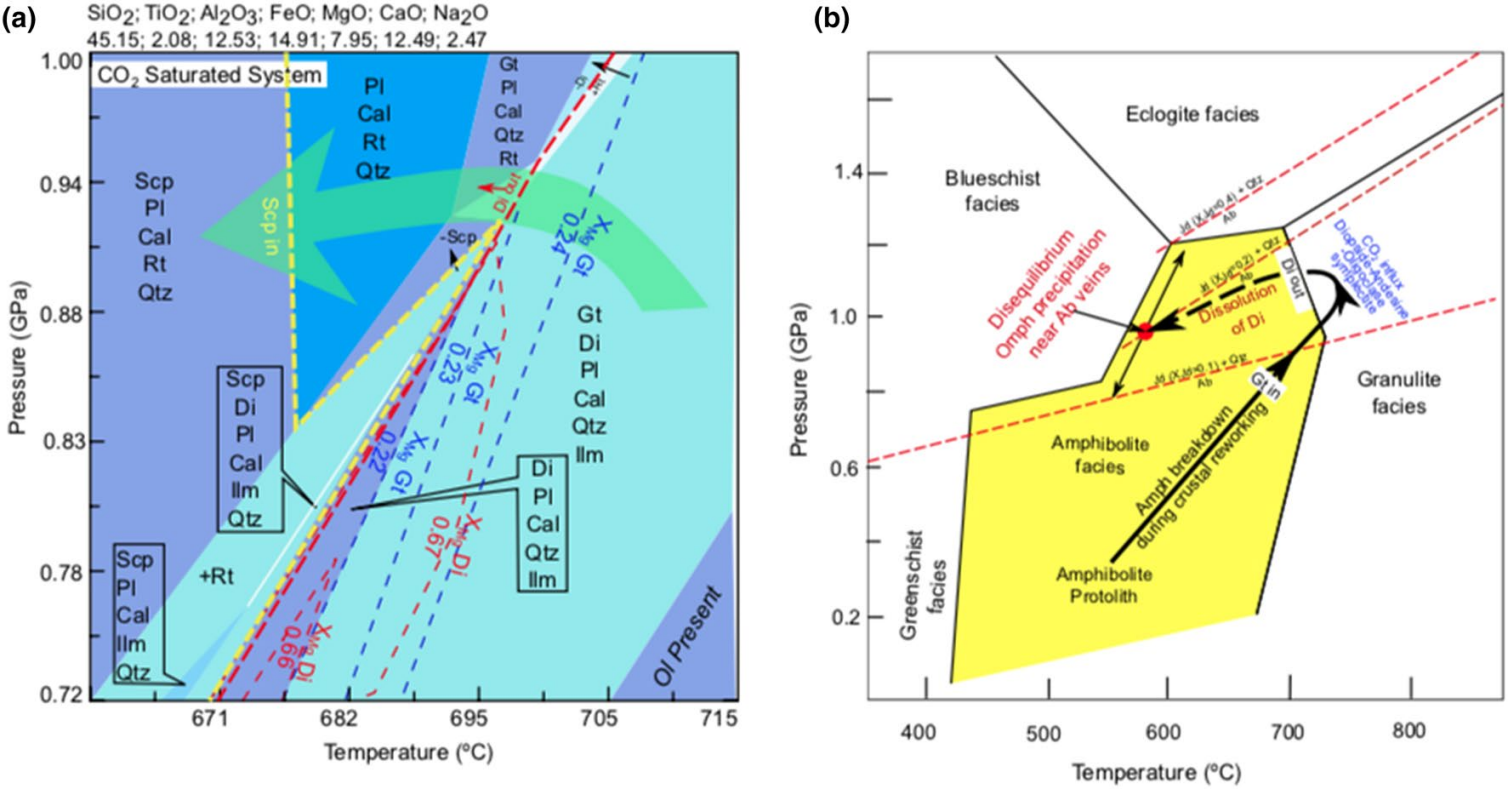
(**a**) A phase diagram created using Perple_X software 6.7.9 version (inbuilt thermodynamic data file^60^) available at http://www.perplex.ethz.ch^59^, and the published Bulk chemistry data of the Bibong amphibolite^37^. The bulk chemistry data (in wt%) is given above the phase diagram. Light green arrow shows stability of scapolite during cooling. (**b**) Phase diagram showing different metamorphic facies conditions^42^ and reaction-1 stability lines^24^ from experimental studies (red lines). The anti-clockwise arrow represents the possible P–T path followed by the amphibolite sample in this study. Di-diopside; Gt-garnet; Pl-plagioclase (andesine-oligoclase); Ab-albite; Jd-jadeite; Qtz-quartz; Cal-calcite; Omph-omphacite; Ilm-Ilmenite; Ol-olivine; Scp-scapolite; Rt-rutile.

Our petrography results show that amphibole is breaking down during the peak metamorphic conditions, where garnet, diopside, plagioclase (andesine-oligoclase) assemblage is present along with scapolite and calcite, and are in textural equilibrium. Presence of calcite (Figs. 1e and 2d) and CO_2_ bearing scapolite (meionite; Figs. 2 and 3) allows us to consider an open CO_2_ saturated system. In an open system environment, a fluid which is out of equilibrium first causes some dissolution of a few monolayers of amphibole (parent) phase and makes the fluid supersaturated with respect to a more stable phases^41^. Such amphibole dissolution along with additional Si and Al components in fluids might provide adequate material to form new minerals during open system metasomatism. In our sample, stable new equilibrium phases formed during metasomatism are garnet, diopside, plagioclase (andesine-oligoclase), calcite and scapolite (Fig. 2b). In our sample, X_Mg_ of garnet varies from 0.17 to 0.23, while X_Mg_ of diopside varies from 0.58 to 0.67 (Extended data Tables S2, S3). The phase diagram result, along with the isopleths of garnet and clinopyroxene, shows that garnet, diopside, plagioclase (andesine-oligoclase), calcite, scapolite and ilmenite/rutile assemblage is stable at temperature ~ 700 ºC and pressure ~ 0.95 GPa (Fig. 5a). Scapolite, appears as scattered sub-micron scale grains in the andesine-oligoclase matrix, is stable towards the cooling direction in the phase diagram.

Albite mainly occurs as thin networks along grain boundaries of andesine-oligoclase matrix and as vein-filling in fractured amphiboles and garnets are texturally in disequilibrium with other phases. Pervasive omphacite formation around albite veins indicate that they too are in disequilibrium with other phases. However immediate association of omphacite and albite indicate that they both are in textural equilibrium at least on 1 mm (milli-meter) scale (Figs. 3d,f, and 4a). This allows us to compare reaction-1 stability lines^24^ from experimental studies with observed P–T conditions. We overlie our phase diagram results and reaction 1 stability lines on published metamorphic facies diagram^42^ (Fig. 5b). The formation of omphacite replacing diopside by disequilibrium coupled dissolution precipitation reactions indicate that diopside was not stable during fluid interaction at lower temperatures. Thus, diopside dissolution occurs at lower temperature. Also, ubiquitous presence of plagioclase represent that the rock is not transformed to eclogite facies, where plagioclase is not stable. In our sample omphacite X_Jd_ content varies from 0.9 to 0.3. Based on above criteria’s a milli-meter length scale albite-omphacite precipitation could have occurred during cooling, where diopside-oligoclase becomes unstable. In mm length scale the reactive fluid enriched in Na could dissolve the unstable parent diopside-oligoclase to form omphacite-albite assemblage. These observations define an anti-clockwise *P*–*T* path during the formation of the observed mineral assemblages (Fig. 5b).

### Formation of disequilibrium garnet-omphacite assemblage in amphibolite facies

The metamorphosed mafic outcrop in this study is mostly composed of amphiboles (> 90%) with local areas (< 10%) carrying higher-grade minerals as symplectites and veins in fractures. Thin-sections from these domains show amphibole breaking down to form garnet-plagioclase (oligoclase-andesine), diopside- plagioclase (oligoclase-andesine) (Figs. 1, 2, 3, 4). Occasional omphacite-albite assemblages replaced diopside-oligoclase near thick albite veins (Figs. 3, 4). The newly formed minerals have no secondary amphiboles formed on their boundaries. The above observations show that the studied rock samples were completely amphibolized prior to the formation of new minerals during metamorphism.

Experimental studies and natural examples both suggest that symplectites could from by the interaction of primary mineral boundaries with reactive fluids^43,44^. We have observed that, minerals such as albite, andesine, calcite and diopside are precipitated along fractures cutting across the amphiboles (Fig. 2a). The vein-filled fractures have albite in composition when they pass through andesine in between the amphibole grains (Fig. 2b). Furthermore, we have noted that albite or andesine-oligoclase is precipitated as vein-fillings in fractures in garnets as well. These observations suggest that, mobile fluids could induce fracturing and generate porosity to allow rapid material transport during metasomatic dissolution–precipitation process^45^. Intense fracturing and precipitation of new minerals as veins in fractures in garnets and amphiboles indicate that fluid induced fracturing and mineral precipitation occurred after garnet-amphibolite to granulite facies conditions. The 258 ± 11 Ma and 225 ± 6.6 Ma Sm–Nd whole rock-garnet internal isochron ages from this rock confirm that garnets might have formed during the Permo-Triassic metamorphic event^26,36,37,39^.

The fluids that caused the alterations or mineral precipitation could act as agents of metasomatism^41,45^. The carrier fluids have imposed coupled dissolution-reprecipitation reactions in the rock and left the system. The mineral assemblage formed during such process could be used to trace back the fluid composition^46^. Amphibole undergoes dissolution to form anhydrous diopside in presence of hot low-H_2_O activity fluids^47^. Calcite and meionite near the thin albite networks in andesine-oligocene matrix in the symplectitic region and as veins in fractures indicate that the system is saturated with CO_2_ fluid (Fig. 2c,d). Dissolution–precipitation reactions caused by CO_2_ fluids could have precipitated the sequence of minerals as vein-filled fractures.

It is important to evaluate the conditions during omphacite is precipitated during metamorphism. The omphacite-albite symplectite has a sieve-like texture, where omphacite appears like a network with dispersed sub-micron size albite grains in them. They occur adjacent to the albite bearing vein-filled fractures. The transition from diopside-oligoclase to omphacite-albite in this region occurs gradually, as we can see from the variation of jadeite in clinopyroxene from 9.5% to maximum 30% (Fig. 4; Extended data Table S8) and X_An_ content in plagioclase from 0.22 to 0.05 (Fig. 4; Extended data Table S5). Such gradual variation in elemental concentration during mineral transformation indicates fluid interfaces, where the fluids selectively dissolve unstable minerals and reprecipitate new ones^41,45,48,49^. Experimental results shows that calcic plagioclase is replaced by albite in aqueous solutions at 600 °C within weeks, where parent plagioclase and product albite are out of equilibrium^50^. Therefore, such gradual variations might represent a coupled dissolution-reprecipitation reaction, where CO_2_ fluids preferentially, in a mm length scale, dissolved diopside-plagioclase symplectite, and the resultant fluid produced the omphacite-albite phases (also see pseudosection chapter above).

Comparing textural and mineral chemistry results with pseudosection output, we infer that the rock went through garnet-amphibolite facies condition initially. At this stage, influx of CO_2_ fluid induced fractures in the earlier formed minerals such as amphibole and garnet. Subsequently, in those fractures, new minerals will precipitate as veins, and also develop symplectites. However, omphacite formation, by replacing diopside, near albite veins does not observed everywhere. Fluids rich in dissolved in Na could have migrated to low stress regions in the outcrop. This is evident from the thin veins of albite (Fig. 2c) in the samples without omphacite and thick veins of albite where diopside dissolution and omphacite formation occurs (Figs. 3d–f, 4). During cooling, the *P*–*T* path approached conditions where omphacite-albite assemblage is stable. The maximum jadeite content in the omphacite in our sample is ~ 30% (Extended data Table S8). Also garnet in the sample TS3 has 56% almandine, 27% grossular and minor (16%) pyrope contents, and there is no X_Mg_ increase towards its rim (Extended data Fig. 3; Extended data Table S3). There is no other mineralogical evidence in this sample to suggest that the omphacite was formed during high-pressure metamorphism. There are many reports of low-pressure albite-omphacite occurrences in ‘garnet-free rocks’ as late veins cutting in amphibolites, greenstones, metagraywaekes^51,52^. However, our study shows that below granulite facies conditions, omphacite occurs in disequilibrium with garnet bearing metamorphosed mafic (gabbroic/basaltic) rocks.

During crustal reworking and deformational events, CO_2_ influx from decarbonation of the nearby or lower crustal carbonate sources induced fractures, and could have produced the observed assemblages in the amphibolite. Symplectitic microstructures could develop in metamorphic rocks during retrogression and/or deformation^53^. Following culmination of the transient fluid activity, the arrested assemblages were exhumed to the surface without further reactions.

### Implications of disequilibrium garnet-omphacite in mafic rocks

Conventionally the presence of omphacite in a crustal rock has been correlated with high pressure tectonic environment, especially if garnet is present and is in equilibrium with omphacite. The problem arises when they are present as relict phases where it is difficult to distinguish whether they were formed in equilibrium or disequilibrium. We provide an important example that garnet and omphacite could form in disequilibrium below granulite facies conditions. If such rocks undergo another metamorphic or hydration event there is a possibility that the rock preserve relict garnet and omphacite. Our results alert that textural associations of eclogite resembling assemblages, without any other supporting evidence for high-pressure metamorphism, should be carefully evaluated before defining convergent tectonics including paleo-suture zones and also arriving at conclusions due to diverse situations such as deep subduction and rapid uplift through subduction channel^54^, metasomatic alterations in the deeper parts of the continental crust^45^, cooling of lower crustal mafic granulite through eclogite facies, which could cause foundering of lower mafic crust into mantle^55,56^, and local over-pressuring due to amphibolization or partial melting during crustal reworking/shearing^57,58^. Our study highlights the important role of syn-metamorphic metasomatism in creating disequilibrium assemblages resembling high pressure metamorphism in mafic rocks.

## Methods

### Electron microprobe (EPMA)

Electron microprobe analyses were conducted on a JEOL JXA-8100 Superprobe, Electron Probe Micro Analyzer (EPMA), housed at the Department of Earth System Sciences, Yonsei University, Seoul, South Korea. Core to rim compositions of important mineral assemblages in TS1, TS2 and TS3 were analyzed. Analytical conditions are accelerating voltage of 20 kV; beam current of 20 nA; counting time of 10 s and an electron beam spot size of 5 μm. Peak position was adjusted to 129.9 for measuring Na content in the omphacite. In the standard position 129.27, Na content and total composition were low. Similarly, slight adjustment has done to measure Mg and Al in garnet, amphibole, diopside and omphacite to obtain maximum count by scanning for peaks before quantitative analysis. Natural and synthetic silicates and oxides supplied by JEOL and ASTIMEX standards Ltd., Canada, were used for calibration. The data were reduced using the ZAF correction procedures supplied by JEOL.

### Phase diagram

Phase diagram modeling using bulk-chemistry of the amphibolite provide a good opportunity to examine the stability of different mineral phases and their compositional changes during the evolution of this particular rock in a pressure temperature space. PERPLE_X 6.7.9 version available at http://www.perplex.ethz.ch^59^ and inbuilt thermodynamic data file^60^ were used for phase diagram calculations. Bulk chemistry (in wt%) of the sample is taken from reference 47 and has SiO_2_; 45.15; TiO_2_; 2.08; Al_2_O_3_; 12.53; FeO; 14.91; MgO; 7.95; CaO; 12.49; Na_2_O; 2.47. The MnO, K_2_O and P_2_O_5_ compositions were not considered due to their low concentrations. The following solid-solution models were used for phase diagram calculation: garnet^61^ (Gt(W)); Diopside^62,63^ (Cpx (h)); plagioclase^64^ (Pl(h)); Ilmenite (IlGkPy (ideal)); calcite^65^ (Cc(AE)); Scapolite (Scp). We have used CO_2_ activity of 1 and X_CO2_ fluid equation of state^60^. The two-dimensional P–T pseudosection was calculated in a window where temperature changes from 660 to 715 ºC on the X-axis and pressure varies from 0.72 to 1 GPa on the Y-axis.

## Supplementary information

Supplementary Figure Legends.

Supplementary Information.
