# Supplementary material for: Eclogite resembling metamorphic disequilibrium assemblage formed through fluid-induced metasomatic reactions
Source: Sci Rep. 2020 Nov 16;10:19869. doi: 10.1038/s41598-020-76999-y (PMC7670418; doi:10.1038/s41598-020-76999-y)
Supplement: Supplementary file 1 — Supplementary Figure Legends. [file 41598_2020_76999_MOESM1_ESM.docx]

**Extended data**

**Extended data Figure 1**: Geologic map. a) Inset box shows a simplified tectonic map of East Asia with the location of the Hongseong area. b) Detailed geologic map (drawn using Adobe Illustrator CS6 V.16.0 supported by Yonsei University; available at: https://adobe.com/products/illustrator) of the Bibong amphibolite area, Hongseong, central southwestern Korean Peninsula with sample locations (Modified after reference 36).

**Extended data Figure 2**: **a–d**, Field photos of the amphibolite with areas/pockets bearing feldspar, garnet and clinopyroxene. White feldspar veins occur along all the fractures, ranging from a few mm to cm in width.

**Extended data Figure 3**: Element compositional maps generated using EPMA facility at Department of Earth System Sciences, Yonsei University, Seoul, South Korea. **a,** MgO compositional map of garnet from TS1 sample. **b**, MnO compositional map of garnet from TS1 sample. The variation of MgO and MnO content (from Extended Data Table S3) are plotted below the respective compositional maps using MS Excel V.16.16; available at https://office.microsoft.com/excel. MnO content marginally increases from core (1.3 wt%) to rim (2.1 wt%).

**Extended data Figure 4**: Schematic representation shows different stages of metamorphism of amphibolite sample; created using Inkscape V.1.0, available at https://inkscape.org. **a,** Stage-1: Amphibolite protolith before metamorphism consist of pristine amphibole laths of ~2 to 5 mm size. **b,** Stage-2: Garnet forms in the garnet-amphibolite facies condition and influx of CO_2_ induce fracturing. **c,** Stage-3: Dissolution of amphibole boundaries in the presence of low H_2_O activity CO_2_ fluid and precipitates symplectites **d & e,** Stage-4: During cooling excess Na and Si dissolved in CO_2_ fluid precipitates albite and omphacite.
