# Supplementary material for: Eclogite resembling metamorphic disequilibrium assemblage formed through fluid-induced metasomatic reactions
Source: Sci Rep. 2020 Nov 16;10:19869. doi: 10.1038/s41598-020-76999-y (PMC7670418; doi:10.1038/s41598-020-76999-y)
Supplement: Supplementary file 2 — Supplementary Information. [file 41598_2020_76999_MOESM2_ESM.pdf]

Eclogite resembling metamorphic disequilibrium assemblage formed through fluid-induced metasomatic reactions

Sanghoon Kwon, Vinod O. Samuel, Yungoo Song, Sung Won Kim, Seung-Ik Park, Yirang Jang, & M. Santosh

Extended Text 1: Mineral Chemistry

Amphibole

Petrographic studies show that amphibole constitutes the ubiquitous mineral in all the samples. We classified the amphiboles following classification scheme of Hawthorne et al. (2012). Amphibole compositions vary among magnesio-hornblende to magnesio-ferri-hornblende in TS2 and pargasite to ferro-pargasite in TS3. Al_2O_3 (~3 wt%), K_2O (~0.5 wt%) and Na_2O (~2 wt%) are slightly higher in the amphiboles associated to omphacite-bearing sample TS3 (Extended data Table 1). The Fe and Mg contents are approximately similar in both amphiboles. The X_{Mg} in amphibole varies from 0.51 to 0.54 in the TS2 and 0.48 to 0.49 in TS3 (Extended data Table 1).

Diopside

In all the analyzed samples, diopside occurs as symplectitic grains formed by breakdown of amphibole and as vein in fractures cutting across amphibole laths (Figs. 1, 2). Both these pyroxenes in TS2 and TS3 samples have diopside composition (Extended data Table 2). The former has ~45.2 percent and the latter has ~47.4 percent wollastonite component. In the TS2 symplectitic diopside, the X_{Mg} varies from 0.59 to 0.64, and X_{Al} of 0.03. Maximum Na_2O content is 0.9 wt%, with a slightly enriched MnO content of ~0.25wt%. In the TS3 sample, symplectitic diopside has X_{Mg} varies from 0.58 to 0.67, and X_{Al} varies from 0.02 to 0.04. In the TS3 sample, symplectitic diopside has relatively enriched Na content compared to that of TS2 sample. Mineral recalculations were done considering all Fe as FeO, and Fe^{+3} was calculated based

on stoichiometry and charge balance. Using this value diopside, aegerine, jadeite percent was calculated using the equation ((Ca) or (Fe^{+3}) or (Na-Fe^{+3}))/(Fe^{+3}+(Na-Fe^{+3}) + Ca) x 100, respectively. The TS3 jadeite and aegerine content is 8.5 and 0 percent, respectively compared to 2.1 percent Jadeite with 0 percent aegerine in TS2.

Diopsidic clinopyroxene formed as vein in the fractures cutting across the amphiboles has X_{Mg} variation from 0.65 to 0.63 with X_{Al} 0.03. The Na_2O content is low (~0.2) compared to symplectitic diopside (Extended data Table 2). The mineral shows wollastonite component of 45.28 percent, and low jadeite content of 1.43 percent with 0 percent Aegerine.

Omphacite

Amphibole-breakdown symplectite textures rarely show gradual transition from diopside-oligoclase symplectite to omphacite-albite symplectite in the TS3 thin-section (Figs. 3, 4). Analysis has been done by fixing points along where the calcic pyroxene alters to sodic pyroxene. The Na content in the pyroxene varies from less than 1 wt% to ~5.5 wt% along the calcic to sodic transformation (Fig. 4, Extended data Table 8). Mineral recalculations were done similar to the method described for diopsidic-clinopyroxene. The jadeite content varies from 9.5 to 28.7 percent and aegerine content from 1.2 to 8.7 along the calcic to sodic alteration (Fig. 4, Extended data Table 8). This low to high jadeite content from calcic to sodic pyroxene is also associated with increase in SiO_2, Al_2O_3 and decrease in FeO, MgO, MnO and CaO (Extended data Table 8).

Garnet

Both TS2 and TS3 thin sections show that breakdown of amphibole led to the formation of garnet-(oligoclase-andesine) assemblage (Fig. 1c, Fig. 3a-c). In the TS2 sample, garnet is 57 % almandine, 25% grossular and only 15 % pyrope content (Extended data Table 3). The X_{Mg} in the garnet is 0.14–0.16, without any major core to rim variation (Extended data Table 3).

This is also evident in the MgO compositional map of garnet from this sample (Extended data Fig. 3a). The MnO content marginally increases from core (1.3 wt%) to rim (2.1 wt%) which is also evident in the MnO compositional map (Extended data Fig. 3b; Extended data Table 3). Similarly, garnet in the sample TS3 has 56 % almandine, 27 % grossular and minor 16 % pyrope content (Extended data Table 3). The X_{Mg} *in this sample varies from 0.13 to 0.2 and MnO variation from core to rim shows a range of 0.59 wt% to 1.08 wt% (Extended data Table 3).*

Feldspar

Both sodic and calcic plagioclase are mainly present in the symplectitic regions and as veins in fractures in garnet and amphibole (Figs. 1, 3, 4). The calcic plagioclase in TS2 has CaO content (~8 to 9 wt%) slightly higher than Na_2O *(6 to 7 wt%). The* X_{An} *in calcic plagioclase in these samples varies from 0.39 to 0.48, representing andesine composition (Extended data Table 4). Similar textures in the TS3 sample has* X_{An} *from 0.22 to 0.3, showing mainly oligoclase composition (Extended data Table 4). Albite mostly forms as vein in fractures within amphibole (Fig. 2a), plagioclase (Fig. 2b), and garnet (Fig. 3) and also as thin networks in the andesine-oligoclase matrix near the symplectite textures (Fig. 2c). Albite in TS2 sample has ~10 wt%* Na_2O*, and show* X_{An} *ranging from 0.09 to 0.12 (Extended data Table 5). In TS3 sample, there is a trend in the variation of* X_{An} *content from 0.22 to 0.05 where diopside-oligoclase transforms to omphacite-albite symplectite (Fig. 4; Extended data Table 5).*

Scapolite and calcite

Scapolite mainly forms in association with thin albite network in the andesine matrix of the symplectitic domain (Fig. 2a, b, c). The mineral occurs as thin parallel flakes with irregular boundary. The scapolite in these samples is mostly meionite in composition with SiO_2 *43 wt%,* Al_2O_3 *27 wt% and CaO 26wt% (Extended data Table 6). Na was not detected, and Cl content*

is also below the detection limit (less than 0.09 wt%). Another important mineral present in these samples is calcite. Similar to scapolite, calcite also precipitates in the symplectitic area and as vein-filling fractures cutting across the amphibole laths. Calcite in these sample has 55.85 wt% CaO and minor FeO of 0.53 and MnO of 0.16 (Extended data Table 7).

Extended Data Figure 1

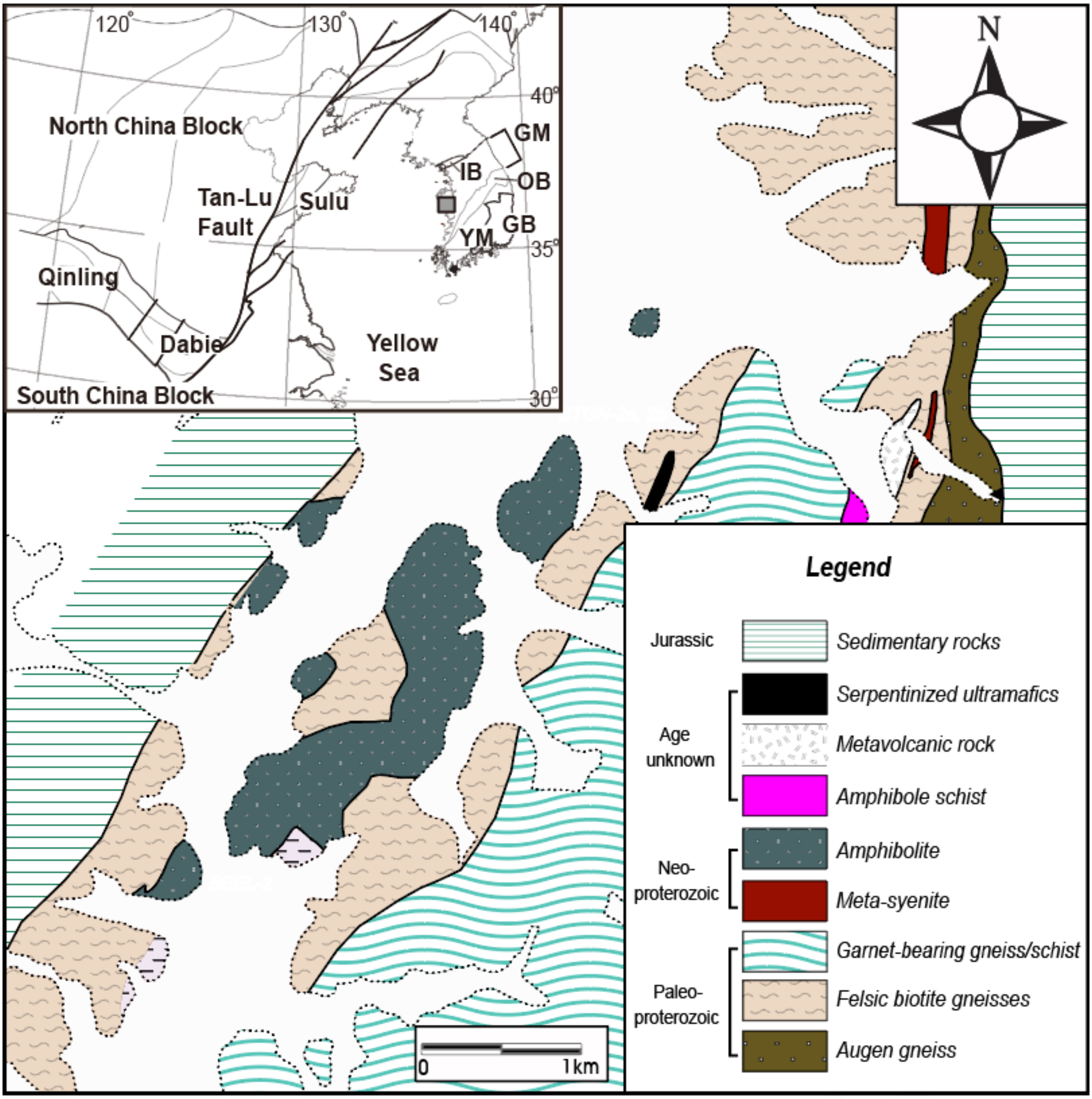

Extended Data Figure 2

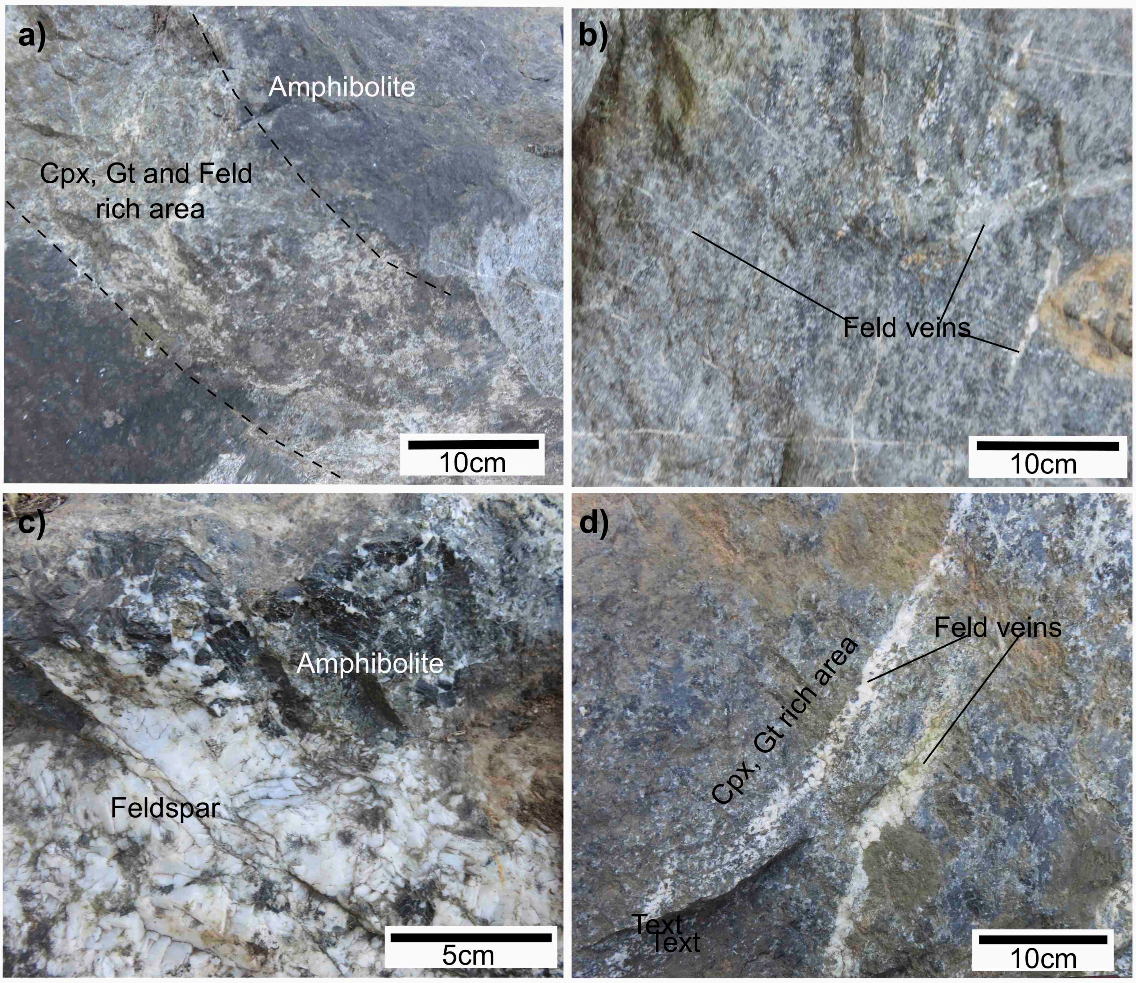

Extended Data Figure 3

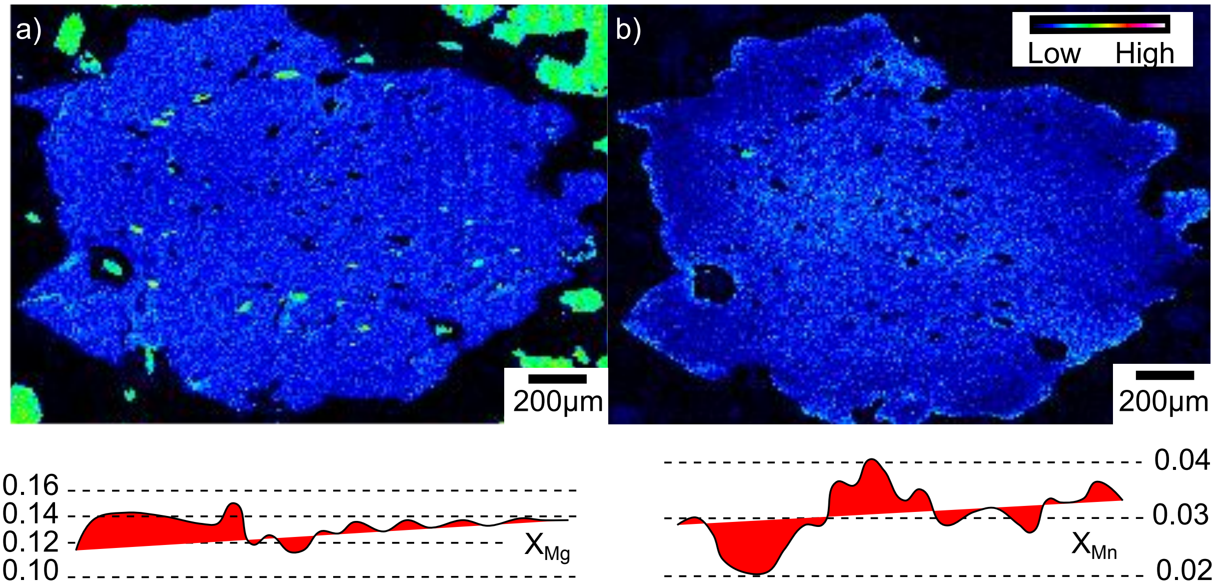

Extended Data Figure 4

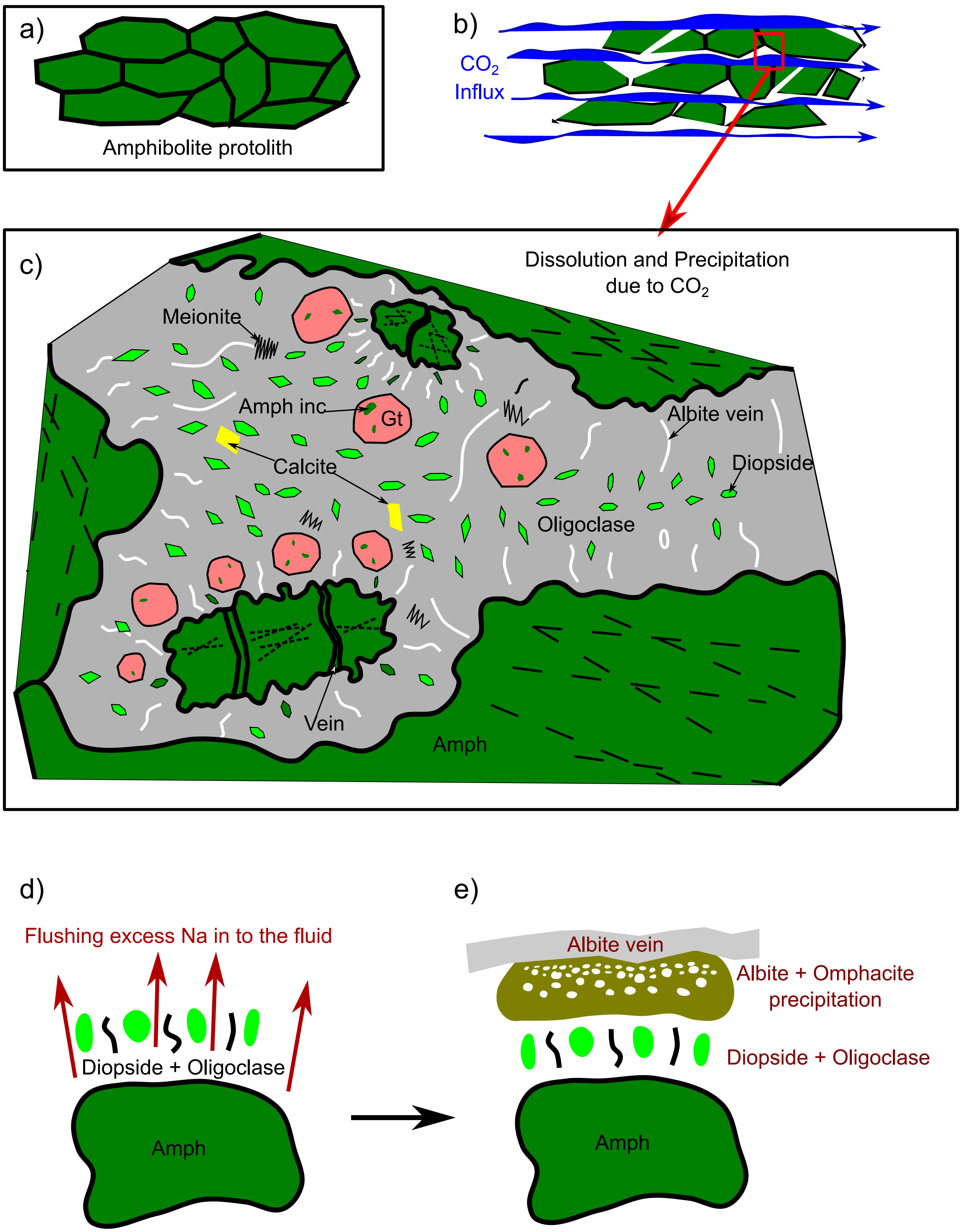

Extended Data Table S1: Representative EPMA results of amphibole (oxide wt%)

Sample	**TS2**												
No.	1	2	3	4	5	6	7	8	9	10	11	12	Average
SiO2	44.52	43.80	43.76	43.88	44.59	43.77	44.04	43.90	44.21	42.78	44.41	43.90	43.96
TiO2	0.74	0.84	0.88	0.86	0.81	0.85	0.81	0.97	0.81	0.96	0.86	0.77	0.84
Al2O3	9.88	10.57	10.68	10.01	11.01	10.41	9.78	10.41	9.69	10.10	10.47	9.65	10.22
Fe2O3*	6.75	8.23	10.22	8.09	9.79	9.17	8.68	8.09	8.42	8.22	8.57	8.40	8.55
FeO	10.19	9.20	7.80	9.39	8.71	8.78	9.12	9.53	9.31	9.72	8.59	9.74	9.17
MnO	0.35	0.30	0.25	0.26	0.27	0.21	0.26	0.30	0.21	0.26	0.21	0.23	0.26
MgO	10.17	10.22	10.30	10.25	10.73	10.29	10.21	10.24	10.46	9.76	10.65	10.12	10.28
CaO	10.81	10.63	10.26	10.56	10.95	10.50	10.54	10.80	10.80	10.59	10.74	10.87	10.67
Na2O	1.04	1.26	1.27	1.25	1.45	1.32	1.05	1.20	1.02	1.15	1.09	0.91	1.17
K2O	0.08	0.08	0.07	0.07	0.07	0.07	0.08	0.06	0.08	0.06	0.06	0.07	0.07
H2O(+)**	1.95	1.97	1.98	1.96	2.03	1.97	1.96	1.98	1.97	1.93	1.99	1.96	1.97
sum	96.47	97.11	97.45	96.57	100.40	97.32	96.50	97.47	96.96	95.54	97.63	96.62	97.17
Structural formula on the basis of O22(OH)2													
Si	6.7470	6.6001	6.5560	6.6513	6.5109	6.5828	6.6758	6.5994	6.6737	6.5822	6.6296	6.6663	6.6225
Al(T)	1.2530	1.3999	1.4440	1.3487	1.4891	1.4172	1.3242	1.4006	1.3263	1.4178	1.3704	1.3337	1.3775
Sum(T)	8.0000	8.0000	8.0000	8.0000	8.0000	8.0000	8.0000	8.0000	8.0000	8.0000	8.0000	8.0000	8.0000
Al(M1-M3)	0.5125	0.4777	0.4417	0.4400	0.4048	0.4280	0.4227	0.4445	0.3973	0.4139	0.4721	0.3937	0.4374
Ti	0.0843	0.0955	0.0986	0.0975	0.0889	0.0958	0.0920	0.1093	0.0918	0.1107	0.0967	0.0880	0.0957
Fe(3+)	0.7693	0.9332	1.1518	0.9231	1.0752	1.0379	0.9896	0.9152	0.9559	0.9511	0.9628	0.9603	0.9693
Fe2+	1.2912	1.1589	0.9772	1.1905	1.0638	1.1041	1.1559	1.1979	1.1747	1.2512	1.0723	1.2376	1.1556
Mn	0.0451	0.0380	0.0311	0.0327	0.0328	0.0264	0.0327	0.0377	0.0267	0.0338	0.0271	0.0295	0.0328
Mg(M1-M3)	2.2977	2.2967	2.2996	2.3162	2.3346	2.3078	2.3071	2.2954	2.3536	2.2394	2.3690	2.2909	2.3092
sum(M1-M3)	5.0000	5.0000	5.0000	5.0000	5.0000	5.0000	5.0000	5.0000	5.0000	5.0000	5.0000	5.0000	5.0000
Mg(M4)	0.0000	0.0000	0.0000	0.0000	0.0000	0.0000	0.0000	0.0000	0.0000	0.0000	0.0000	0.0000	0.0000
Ca	1.7562	1.7167	1.6477	1.7149	1.7133	1.6919	1.7123	1.7392	1.7473	1.7460	1.7173	1.7688	1.7225
Na(M4)	0.2438	0.2833	0.3523	0.2851	0.2867	0.3081	0.2877	0.2608	0.2527	0.2539	0.2827	0.2312	0.2775
sum(M4)	2.0000	2.0000	2.0000	2.0000	2.0000	2.0000	2.0000	2.0000	2.0000	2.0000	2.0000	2.0000	2.0000
Na	0.0630	0.0849	0.0158	0.0820	0.1247	0.0753	0.0200	0.0884	0.0446	0.0900	0.0328	0.0379	0.0634
K	0.0157	0.0156	0.0136	0.0128	0.0127	0.0132	0.0149	0.0123	0.0146	0.0126	0.0110	0.0128	0.0135
Sum(A)	0.0786	0.1004	0.0293	0.0948	0.1373	0.0886	0.0349	0.1007	0.0593	0.1025	0.0438	0.0507	0.0768
OH-	1.9679	1.9809	1.9762	1.9808	1.9807	1.9792	1.9807	1.9824	1.9830	1.9829	1.9810	1.9844	1.9800
Mg/(Mg+Fe2+)	0.6402	0.6646	0.7018	0.6605	0.6870	0.6764	0.6662	0.6571	0.6671	0.6416	0.6884	0.6493	0.6665

magnesio-hornblende

Sample	TS3								
No.	1	2	3	4	5	6	7	8	Average
SiO2	41.49	41.85	42.00	42.10	42.26	41.52	41.89	42.25	41.92
TiO2	0.65	0.83	0.69	0.69	0.90	1.65	0.98	0.80	0.90
Al2O3	14.17	14.11	14.03	13.52	13.35	13.22	13.68	13.26	13.67
Fe2O3*	4.10	3.19	2.83	2.18	1.62	1.37	2.53	1.94	2.47
FeO	13.70	14.33	14.77	15.04	15.39	15.88	15.05	15.75	14.99
MnO	0.13	0.15	0.14	0.14	0.15	0.18	0.13	0.18	0.15
MgO	9.25	9.11	9.09	9.15	9.14	9.02	9.11	9.08	9.12
CaO	10.94	10.92	10.89	10.95	10.88	11.01	11.01	11.12	10.97
Na2O	3.22	3.15	3.33	3.20	3.24	3.25	3.19	3.22	3.23
K2O	0.57	0.57	0.57	0.55	0.53	0.56	0.57	0.56	0.56
H2O(+)**	1.99	2.00	2.00	1.99	1.99	1.98	2.00	2.00	1.99
sum	100.19	100.21	100.34	99.52	99.43	99.63	100.13	100.14	99.95
Si	6.1916	6.2394	6.2619	6.3250	6.3512	6.2574	6.2646	6.3280	6.2773
Al(T)	1.8084	1.7606	1.7381	1.6750	1.6488	1.7426	1.7354	1.6720	1.7227
Sum(T)	8.0000	8.0000	8.0000	8.0000	8.0000	8.0000	8.0000	8.0000	8.0000
Al(M1-M3)	0.6842	0.7184	0.7266	0.7180	0.7156	0.6058	0.6760	0.6681	0.6892
Ti	0.0726	0.0935	0.0768	0.0775	0.1018	0.1874	0.1102	0.0905	0.1012
Fe(3+)	0.4599	0.3573	0.3169	0.2469	0.1827	0.1555	0.2851	0.2181	0.2781
Fe2+	1.7100	1.7867	1.8418	1.8901	1.9341	2.0013	1.8823	1.9729	1.8771
Mn	0.0159	0.0186	0.0177	0.0181	0.0187	0.0229	0.0165	0.0227	0.0189
Mg(M1-M3)	2.0573	2.0255	2.0202	2.0493	2.0471	2.0272	2.0300	2.0276	2.0355
sum(M1-M3)	5.0000	5.0000	5.0000	5.0000	5.0000	5.0000	5.0000	5.0000	5.0000
Mg(M4)	0.0000	0.0000	0.0000	0.0000	0.0000	0.0000	0.0000	0.0000	0.0000
Ca	1.7499	1.7449	1.7393	1.7628	1.7527	1.7776	1.7637	1.7842	1.7594
Na(M4)	0.2501	0.2550	0.2606	0.2372	0.2473	0.2224	0.2363	0.2158	0.2406
sum(M4)	2.0000	2.0000	2.0000	2.0000	2.0000	2.0000	2.0000	2.0000	2.0000
Na	0.6802	0.6555	0.7022	0.6940	0.6978	0.7262	0.6899	0.7206	0.6957
K	0.1081	0.1075	0.1084	0.1054	0.1016	0.1071	0.1084	0.1061	0.1066
Sum(A)	0.7883	0.7629	0.8106	0.7994	0.7994	0.8333	0.7983	0.8266	0.8023
OH-	1.9809	1.9900	1.9910	1.9930	1.9949	1.9956	1.9920	1.9939	1.9914
Mg/(Mg+Fe2+)	0.5461	0.5313	0.5231	0.5202	0.5142	0.5032	0.5189	0.5068	0.5202

Pargasite

Fe2O3* was calculated by stoichiometry assuming only Ca and Na in M4 site.
H2O(+)** was calculated by stoichiometry assuming the number of (OH)- is 2.0

Extended Data Table S2: Representative EPMA results of diopside (oxide wt%)

Sample Label	**TS2**										
No	1	2	3	4	5	6	7	8	9	10	Average
SiO2	54.61	53.13	54.88	54.11	54.15	54.81	52.43	54.29	54.33	54.38	54.11
TiO2	0.09	0.04	0.07	0.05	0.06	0.03	0.64	0.37	0.09	0.04	0.15
Al2O3	1.30	1.28	1.35	1.34	1.27	1.33	2.72	1.40	1.32	1.18	1.45
Cr2O3	0.00	0.00	0.00	0.00	0.04	0.00	0.03	0.01	0.04	0.01	0.01
FeO	12.62	12.51	11.53	11.99	12.22	12.20	12.56	12.40	12.25	11.37	12.16
MnO	0.29	0.24	0.23	0.25	0.24	0.30	0.24	0.22	0.32	0.19	0.25
MgO	11.61	11.67	11.61	11.41	11.57	11.53	10.11	10.94	11.44	11.33	11.32
CaO	20.64	20.82	21.02	20.72	20.52	20.70	20.77	20.10	21.13	22.01	20.84
Na2O	0.15	0.14	0.18	0.15	0.15	0.13	0.90	0.42	0.16	0.14	0.25
K2O	0.00	0.00	0.00	0.00	0.00	0.00	0.00	0.00	0.00	0.00	0.00
sum	101.29	99.81	100.86	100.01	100.23	101.04	100.38	100.15	101.07	100.64	100.55
	Structural formula on the basis of 6 oxygens										
Si	2.0226	2.0040	2.0316	2.0260	2.0244	2.0303	1.9716	2.0306	2.0182	2.0241	2.0184
Al(T)	0.0000	0.0000	0.0000	0.0000	0.0000	0.0000	0.0284	0.0000	0.0000	0.0000	0.0000
Sum(T)	2.0226	2.0040	2.0316	2.0260	2.0244	2.0303	2.0000	2.0306	2.0182	2.0241	2.0184
Al(O)	0.0567	0.0568	0.0587	0.0590	0.0558	0.0581	0.0920	0.0617	0.0579	0.0519	0.0636
Ti	0.0024	0.0010	0.0020	0.0014	0.0017	0.0007	0.0181	0.0103	0.0024	0.0012	0.0041
Cr(3+)	0.0000	0.0000	0.0000	0.0000	0.0032	0.0000	0.0025	0.0008	0.0035	0.0004	0.0010
Fe	0.3907	0.3945	0.3568	0.3753	0.3822	0.3781	0.3949	0.3878	0.3804	0.3539	0.3794
Mn	0.0090	0.0075	0.0073	0.0079	0.0076	0.0094	0.0075	0.0069	0.0099	0.0058	0.0079
Mg	0.6409	0.6563	0.6408	0.6370	0.6450	0.6368	0.5665	0.6099	0.6333	0.6285	0.6295
Ca	0.8190	0.8413	0.8335	0.8311	0.8221	0.8218	0.8367	0.8053	0.8408	0.8780	0.8330
Na	0.0106	0.0105	0.0127	0.0110	0.0105	0.0093	0.0658	0.0307	0.0112	0.0100	0.0182
K	0.0000	0.0000	0.0000	0.0000	0.0000	0.0000	0.0000	0.0000	0.0000	0.0000	0.0000
Sum	1.9293	1.9679	1.9118	1.9227	1.9282	1.9142	1.9841	1.9134	1.9393	1.9297	1.9368
X_{Mg}	0.62	0.62	0.64	0.63	0.63	0.63	0.59	0.61	0.62	0.64	0.62

Sample Label	**TS2-Vein**												
No	1	2	3	4	5	6	7	8	9	10	11	12	Average
SiO2	53.45	54.17	54.27	54.20	54.14	54.45	52.07	53.39	54.03	52.06	53.21	53.36	53.57
TiO2	0.03	0.08	0.05	0.05	0.06	0.05	0.05	0.02	0.07	0.05	0.05	0.07	0.05
Al2O3	1.36	1.37	1.43	1.36	1.38	1.38	1.38	1.32	1.37	1.37	1.43	1.13	1.36
Cr2O3	0.00	0.00	0.04	0.02	0.00	0.02	0.01	0.03	0.03	0.01	0.03	0.02	0.02
FeO	11.86	11.72	11.86	12.21	11.73	12.06	12.06	12.03	11.62	11.29	11.84	11.98	11.86
MnO	0.25	0.22	0.28	0.30	0.26	0.23	0.25	0.24	0.29	0.23	0.31	0.31	0.26
MgO	11.56	11.85	11.86	11.82	11.86	11.83	12.33	11.90	11.85	12.01	11.52	11.71	11.84
CaO	21.25	21.29	21.15	21.45	21.19	21.29	21.17	20.98	21.43	21.88	21.02	21.36	21.29
Na2O	0.17	0.16	0.22	0.16	0.17	0.13	0.19	0.16	0.14	0.19	0.21	0.15	0.17
K2O													
sum	99.92	100.85	101.15	101.57	100.78	101.44	99.52	100.07	100.83	99.09	99.62	100.09	100.41
Si	2.0090	2.0127	2.0113	2.0058	2.0130	2.0130	1.9746	2.0044	2.0092	1.9796	2.0063	2.0058	2.0038
Al(T)	0.0000	0.0000	0.0000	0.0000	0.0000	0.0000	0.0254	0.0000	0.0000	0.0204	0.0000	0.0000	0.0000
Sum(T)	2.0090	2.0127	2.0113	2.0058	2.0130	2.0130	2.0000	2.0044	2.0092	2.0000	2.0063	2.0058	2.0038
Al(O)	0.0601	0.0600	0.0626	0.0595	0.0603	0.0603	0.0363	0.0584	0.0600	0.0412	0.0637	0.0499	0.0598
Ti	0.0010	0.0022	0.0013	0.0013	0.0016	0.0014	0.0015	0.0005	0.0020	0.0013	0.0014	0.0020	0.0014
Cr(3+)	0.0000	0.0000	0.0030	0.0017	0.0002	0.0013	0.0006	0.0021	0.0024	0.0009	0.0024	0.0013	0.0013
Fe	0.3728	0.3642	0.3676	0.3780	0.3648	0.3728	0.3824	0.3778	0.3615	0.3590	0.3734	0.3767	0.3709
Mn	0.0080	0.0069	0.0086	0.0093	0.0081	0.0073	0.0080	0.0076	0.0091	0.0074	0.0100	0.0098	0.0083
Mg	0.6476	0.6562	0.6554	0.6521	0.6575	0.6520	0.6973	0.6660	0.6567	0.6807	0.6475	0.6564	0.6604
Ca	0.8555	0.8474	0.8398	0.8505	0.8440	0.8433	0.8603	0.8440	0.8541	0.8912	0.8493	0.8604	0.8532
Na	0.0121	0.0112	0.0157	0.0115	0.0120	0.0093	0.0140	0.0119	0.0100	0.0143	0.0150	0.0112	0.0123
K	0.0000	0.0000	0.0000	0.0000	0.0000	0.0000	0.0000	0.0000	0.0000	0.0000	0.0000	0.0000	0.0000
Sum	1.9571	1.9481	1.9541	1.9639	1.9485	1.9477	2.0003	1.9684	1.9557	1.9958	1.9626	1.9676	1.9678
X_{Mg}	0.63	0.64	0.64	0.63	0.64	0.64	0.65	0.64	0.64	0.65	0.63	0.64	0.64

Sample Label	TS3										
No	1	2	3	4	5	6	7	8	9	10	Average
SiO2	53.99	53.33	53.25	53.43	53.63	54.05	53.16	53.56	54.10	54.21	53.67
TiO2	0.13	0.11	0.09	0.11	0.13	0.32	0.01	0.36	0.41	0.25	0.19
Al2O3	1.85	1.96	1.98	1.86	1.13	1.84	1.97	1.61	1.86	1.09	1.71
Cr2O3	0.02	0.01	0.02	0.02	0.01	0.01	0.02	0.08	0.00	0.04	0.02
FeO	10.36	10.86	10.87	10.55	11.56	11.11	11.13	11.93	11.97	11.76	11.21
MnO	0.11	0.07	0.03	0.07	0.06	0.11	0.16	0.06	0.07	0.08	0.08
MgO	10.28	11.57	11.31	11.57	12.03	10.49	12.47	9.47	9.33	9.75	10.82
CaO	21.22	21.25	21.37	21.34	21.58	21.79	21.62	21.93	21.09	21.06	21.42
Na2O	1.59	1.39	1.45	1.42	0.46	1.04	0.26	1.17	1.14	1.09	1.10
K2O											
sum	99.54	100.54	100.36	100.36	100.58	100.76	100.78	100.14	99.97	99.33	100.24
Si	2.0270	1.9900	1.9915	1.9948	2.0028	2.0115	1.9778	2.0165	2.0316	2.0479	2.0091
Al(T)	0.0000	0.0100	0.0085	0.0052	0.0000	0.0000	0.0222	0.0000	0.0000	0.0000	0.0000
Sum(T)	2.0270	2.0000	2.0000	2.0000	2.0028	2.0115	2.0000	2.0165	2.0316	2.0479	2.0091
Al(O)	0.0817	0.0760	0.0787	0.0765	0.0499	0.0806	0.0642	0.0716	0.0824	0.0485	0.0756
Ti	0.0036	0.0031	0.0025	0.0031	0.0036	0.0089	0.0002	0.0101	0.0116	0.0070	0.0054
Cr(3+)	0.0014	0.0008	0.0013	0.0019	0.0004	0.0011	0.0013	0.0059	0.0000	0.0030	0.0017
Fe	0.3253	0.3388	0.3400	0.3293	0.3609	0.3459	0.3463	0.3755	0.3760	0.3716	0.3509
Mn	0.0036	0.0023	0.0009	0.0023	0.0018	0.0036	0.0049	0.0019	0.0022	0.0027	0.0026
Mg	0.5754	0.6434	0.6308	0.6439	0.6694	0.5817	0.6914	0.5314	0.5221	0.5488	0.6040
Ca	0.8536	0.8494	0.8564	0.8536	0.8635	0.8691	0.8618	0.8846	0.8484	0.8526	0.8593
Na	0.1155	0.1008	0.1048	0.1029	0.0334	0.0748	0.0187	0.0851	0.0826	0.0801	0.0798
K	0.0000	0.0000	0.0000	0.0000	0.0000	0.0000	0.0000	0.0000	0.0000	0.0000	0.0000
Sum	1.9600	2.0146	2.0154	2.0135	1.9828	1.9658	1.9888	1.9661	1.9252	1.9144	1.9794
X_{Mg}	0.64	0.66	0.65	0.66	0.65	0.63	0.67	0.59	0.58	0.60	0.63

Extended Data Table S3: Representative EPMA results of garnet (wt%)

Sample Label	TS2														
	core	core	core	towards rim	towards rim	towards rim	towards rim	towards rim	towards rim	towards rim	towards rim	towards rim	rim	rim	rim	
No.	1	2	3	4	5	6	7	8	9	10	11	12	13	14	15	Average
SiO2	37.18	37.58	37.66	37.35	37.52	37.37	37.50	37.51	37.73	37.59	37.58	37.61	37.30	37.30	37.55	37.49
TiO2	0.06	0.06	0.11	0.05	0.05	0.03	0.05	0.01	0.06	0.05	0.04	0.03	0.02	0.02	0.02	0.04
Al2O3	21.80	22.03	21.89	22.04	22.00	22.27	21.84	22.00	22.06	21.88	21.95	22.14	22.10	22.01	22.01	22.00
Cr2O3	0.02	0.01	0.01	0.01	0.03	0.01	0.01	0.00	0.03	0.00	0.03	0.02	0.02	0.01	0.04	0.02
Fe2O3(total)	28.69	29.04	27.91	27.99	27.90	28.60	28.20	28.34	28.34	28.49	29.85	29.08	28.76	29.97	30.16	28.76
Fe2O3*	0.05	0.00	0.00	0.00	0.00	0.00	0.00	0.00	0.00	0.00	0.00	0.07	0.00	0.00	0.00	0.00
FeO	25.77	26.13	25.11	25.19	25.10	25.73	25.37	25.50	25.50	25.64	26.86	26.10	25.88	26.97	27.14	25.87
MnO	1.30	1.67	1.50	1.37	1.46	1.31	1.51	1.26	1.44	1.38	1.75	1.47	1.37	2.16	2.12	1.54
MgO	3.59	3.73	3.50	3.62	3.60	3.67	3.68	3.71	3.57	3.66	3.99	3.76	3.71	3.82	3.93	3.70
CaO	9.39	8.85	9.32	9.48	9.38	9.37	9.25	9.22	9.58	9.39	7.66	9.24	8.81	7.30	6.87	8.87
sum	99.17	100.06	99.10	99.10	99.15	99.77	99.21	99.20	99.97	99.60	99.86	100.43	99.20	99.59	99.68	99.54
	Structural formula on the basis of 12 oxygens															
Si	2.9519	2.9567	2.9799	2.9579	2.9682	2.9439	2.9685	2.9669	2.9648	2.9663	2.9638	2.9481	2.9545	2.9555	2.9683	2.9611
Al(IV)	0.0481	0.0433	0.0201	0.0421	0.0318	0.0561	0.0315	0.0331	0.0352	0.0337	0.0362	0.0519	0.0455	0.0445	0.0317	0.0389
sum(T)	3.0000	3.0000	3.0000	3.0000	3.0000	3.0000	3.0000	3.0000	3.0000	3.0000	3.0000	3.0000	3.0000	3.0000	3.0000	3.0000
Al(total)	2.0398	2.0426	2.0409	2.0575	2.0515	2.0681	2.0374	2.0505	2.0427	2.0351	2.0400	2.0454	2.0633	2.0553	2.0507	2.0481
Al(VI)	1.9918	1.9993	2.0208	2.0154	2.0197	2.0120	2.0059	2.0173	2.0075	2.0014	2.0038	1.9935	2.0178	2.0109	2.0190	2.0092
Ti	0.0037	0.0034	0.0066	0.0027	0.0029	0.0020	0.0030	0.0004	0.0037	0.0031	0.0025	0.0015	0.0011	0.0010	0.0012	0.0026
Cr	0.0015	0.0007	0.0006	0.0006	0.0019	0.0007	0.0004	0.0000	0.0018	0.0000	0.0021	0.0009	0.0013	0.0006	0.0026	0.0011
Fe(3+)	0.0030	0.0000	0.0000	0.0000	0.0000	0.0000	0.0000	0.0000	0.0000	0.0000	0.0000	0.0041	0.0000	0.0000	0.0000	0.0000
sum(Y)	2.0000	2.0034	2.0280	2.0188	2.0246	2.0147	2.0094	2.0178	2.0130	2.0046	2.0084	2.0000	2.0202	2.0124	2.0228	2.0128
Fe(2+)	1.7111	1.7197	1.6614	1.6683	1.6609	1.6955	1.6800	1.6870	1.6757	1.6919	1.7712	1.7113	1.7144	1.7872	1.7941	1.7092
Mn	0.0874	0.1113	0.1006	0.0917	0.0980	0.0876	0.1010	0.0842	0.0958	0.0923	0.1166	0.0976	0.0917	0.1448	0.1422	0.1028
Mg	0.4247	0.4379	0.4127	0.4271	0.4240	0.4308	0.4344	0.4369	0.4183	0.4303	0.4694	0.4399	0.4380	0.4513	0.4630	0.4359
Ca	0.7991	0.7460	0.7901	0.8044	0.7948	0.7912	0.7848	0.7816	0.8063	0.7939	0.6470	0.7763	0.7477	0.6198	0.5816	0.7510
sum(X)	3.0222	3.0148	2.9647	2.9916	2.9776	3.0051	3.0001	2.9897	2.9962	3.0084	3.0043	3.0251	2.9919	3.0031	2.9810	2.9989
Almandine	56.62	57.04	56.04	55.77	55.78	56.42	56.00	56.43	55.93	56.24	58.96	56.57	57.30	59.51	60.19	56.99
Pyrope	14.05	14.52	13.92	14.28	14.24	14.34	14.48	14.61	13.96	14.30	15.63	14.54	14.64	15.03	15.53	14.54
grossular	26.33	24.69	26.55	26.84	26.63	26.29	26.11	26.14	26.84	26.35	21.49	25.58	24.96	20.62	19.47	25.00
spessartine	2.89	3.69	3.39	3.07	3.29	2.92	3.37	2.82	3.20	3.07	3.88	3.23	3.07	4.82	4.77	3.43
uvarovite	0.02	0.01	0.01	0.01	0.03	0.01	0.01	0.00	0.02	0.00	0.02	0.01	0.02	0.01	0.02	0.01
andradite	0.04	0.00	0.00	0.00	0.00	0.00	0.00	0.00	0.00	0.00	0.00	0.05	0.00	0.00	0.00	0.00
	99.95	99.96	99.91	99.96	99.96	99.97	99.96	99.99	99.95	99.96	99.97	99.98	99.99	99.99	99.99	99.97
X_{Mg}	0.20	0.20	0.20	0.20	0.20	0.20	0.21	0.21	0.20	0.20	0.21	0.20	0.20	0.20	0.21	0.20

Sample Label	TS3															
	core	core	core	towards rim	towards rim	towards rim	towards rim	towards rim	towards rim	towards rim	towards rim	towards rim	rim	rim	rim	
No.	1	2	3	4	5	6	7	8	9	10	11	12	13	14	15	Average
SiO2	38.27	38.68	38.15	38.09	38.28	38.17	38.01	38.08	38.32	38.80	38.88	38.97	38.34	38.37	38.18	38.37
TiO2	0.04	0.02	0.04	0.02	0.07	0.07	0.15	0.04	0.05	0.02	0.10	0.11	0.06	0.02	0.06	0.06
Al2O3	22.36	21.84	21.71	21.88	22.23	21.89	21.55	22.03	21.65	22.10	21.87	22.07	21.89	22.09	21.39	21.90
Cr2O3	0.01	0.06	0.02	0.02	0.00	0.02	0.02	0.01	0.00	0.00	0.01	0.01	0.04	0.03	0.04	0.02
Fe2O3(total)	28.35	28.37	28.63	28.29	28.48	29.11	27.68	28.58	28.91	28.45	27.97	27.82	28.29	28.39	28.17	28.37
Fe2O3*	0.00	0.00	0.00	0.08	0.00	0.27	1.16	0.11	0.28	0.00	0.00	0.00	0.00	0.00	0.56	0.00
FeO	25.51	25.52	25.76	25.38	25.63	25.95	23.86	25.62	25.76	25.60	25.17	25.03	25.45	25.55	24.85	25.52
MnO	0.59	0.61	0.66	0.58	0.59	0.63	0.78	0.65	0.61	0.59	0.55	0.56	0.49	0.49	1.08	0.63
MgO	3.57	4.35	3.32	4.39	4.22	4.37	5.32	4.49	4.16	4.24	4.13	2.96	2.97	4.02	4.51	4.07
CaO	9.36	9.36	9.57	9.57	9.78	9.30	9.66	9.35	9.55	9.58	10.15	10.42	10.44	9.61	9.30	9.67
sum	99.73	100.45	99.23	100.00	100.80	100.64	100.40	100.36	100.35	100.93	100.87	100.14	99.69	100.18	99.91	100.25
Si	2.9950	3.0055	3.0104	2.9772	2.9698	2.9708	2.9508	2.9674	2.9902	2.9999	3.0072	3.0344	3.0101	2.9910	2.9879	2.9924
Al(IV)	0.0050	0.0000	0.0000	0.0228	0.0302	0.0292	0.0492	0.0326	0.0098	0.0001	0.0000	0.0000	0.0000	0.0090	0.0121	0.0076
sum(T)	3.0000	3.0055	3.0104	3.0000	3.0000	3.0000	3.0000	3.0000	3.0000	3.0000	3.0072	3.0344	3.0101	3.0000	3.0000	3.0000
Al(total)	2.0626	1.9996	2.0192	2.0156	2.0326	2.0078	1.9716	2.0238	1.9906	2.0140	1.9938	2.0251	2.0259	2.0298	1.9730	2.0131
Al(VI)	2.0576	1.9996	2.0192	1.9928	2.0024	1.9786	1.9224	1.9912	1.9808	2.0139	1.9938	2.0251	2.0259	2.0208	1.9609	2.0055
Ti	0.0023	0.0011	0.0024	0.0012	0.0041	0.0041	0.0088	0.0021	0.0028	0.0010	0.0061	0.0066	0.0037	0.0012	0.0037	0.0034
Cr	0.0009	0.0035	0.0014	0.0011	0.0000	0.0014	0.0011	0.0005	0.0000	0.0000	0.0009	0.0004	0.0022	0.0021	0.0026	0.0012
Fe(3+)	0.0000	0.0000	0.0000	0.0049	0.0000	0.0159	0.0676	0.0062	0.0164	0.0000	0.0000	0.0000	0.0000	0.0000	0.0328	0.0000
sum(Y)	2.0608	2.0041	2.0230	2.0000	2.0065	2.0000	2.0000	2.0000	2.0000	2.0150	2.0007	2.0320	2.0318	2.0241	2.0000	2.0101
Fe(2+)	1.6697	1.6586	1.7001	1.6593	1.6632	1.6891	1.5494	1.6697	1.6809	1.6558	1.6280	1.6297	1.6715	1.6655	1.6266	1.6647
Mn	0.0394	0.0403	0.0442	0.0382	0.0388	0.0413	0.0515	0.0428	0.0402	0.0389	0.0358	0.0372	0.0328	0.0325	0.0717	0.0417
Mg	0.4167	0.5037	0.3900	0.5116	0.4881	0.5066	0.6156	0.5217	0.4838	0.4891	0.4764	0.3437	0.3473	0.4674	0.5261	0.4729
Ca	0.7844	0.7796	0.8090	0.8018	0.8131	0.7755	0.8038	0.7810	0.7986	0.7933	0.8413	0.8693	0.8786	0.8024	0.7798	0.8077
sum(X)	2.9101	2.9822	2.9434	3.0108	3.0032	3.0125	3.0202	3.0153	3.0035	2.9771	2.9815	2.8799	2.9302	2.9678	3.0042	2.9870
Almandine	57.37	55.62	57.76	55.11	55.38	56.07	51.30	55.38	55.97	55.62	54.60	56.59	57.04	56.12	54.14	55.73
Pyrope	14.32	16.89	13.25	16.99	16.25	16.82	20.38	17.30	16.11	16.43	15.98	11.93	11.85	15.75	17.51	15.83
grossular	26.91	26.08	27.43	26.53	27.02	25.47	25.58	25.79	26.33	26.63	28.12	30.08	29.90	26.99	25.45	26.98
spessartine	1.35	1.35	1.50	1.27	1.29	1.37	1.70	1.42	1.34	1.31	1.20	1.29	1.12	1.09	2.39	1.40
uvarovite	0.01	0.05	0.02	0.01	0.00	0.02	0.01	0.01	0.00	0.00	0.01	0.01	0.03	0.03	0.03	0.02
andradite	0.00	0.00	0.00	0.07	0.00	0.21	0.90	0.08	0.22	0.00	0.00	0.00	0.00	0.00	0.43	0.00
	99.97	99.99	99.97	99.98	99.94	99.95	99.88	99.97	99.96	99.99	99.91	99.90	99.95	99.98	99.95	99.95
X_{Mg}	0.20	0.23	0.19	0.24	0.23	0.23	0.28	0.24	0.22	0.23	0.23	0.17	0.17	0.22	0.24	0.22

Fe2O3* was calculated by assuming the total number of cations in Y-site is 2.0.

Extended Data Table S4: Representative EPMA results of andesine-oligoclase (wt%)

Sample Label	TS2										TS3									
No.	1	2	3	4	5	6	7	8	9	10	1	2	3	4	5	6	7	8	9	10
SiO2	56.77	57.64	56.53	56.16	55.41	55.40	55.34	55.81	55.85	57.01	62.17	62.29	63.38	62.75	62.03	63.33	62.50	64.23	61.65	61.84
Al2O3	26.79	26.97	26.21	27.45	27.76	27.27	28.21	27.97	27.54	26.63	24.11	24.28	23.38	24.11	24.65	24.11	24.48	23.39	24.73	24.66
FeO	0.07	0.08	0.03	0.06	0.02	0.09	0.12	0.08	0.06	0.06	0.19	0.09	0.16	0.15	0.13	0.16	0.20	0.20	0.26	0.38
CaO	7.92	7.95	7.84	8.75	9.35	8.87	9.55	9.23	8.87	7.67	5.69	6.03	4.93	5.53	6.06	5.52	6.11	4.67	6.46	6.27
BaO	0.00	0.00	0.00	0.00	0.01	0.00	0.00	0.01	0.01	0.00	0.01	0.03	0.01	0.02	0.02	0.00	0.00	0.01	0.01	0.01
Na2O	6.70	6.57	6.49	6.31	5.63	6.07	5.90	5.93	6.06	6.70	8.80	8.46	8.85	8.85	8.41	8.73	8.37	9.14	8.36	8.12
K2O	0.05	0.04	0.06	0.06	0.05	0.05	0.04	0.05	0.05	0.05	0.08	0.12	0.15	0.11	0.09	0.12	0.09	0.17	0.09	0.07
sum	98.31	99.23	97.15	98.80	98.22	97.75	99.14	99.07	98.44	98.13	101.03	101.29	100.85	101.51	101.40	101.97	101.75	101.81	101.57	101.35
	Structural formula on the basis of 8 oxygens																			
Si	2.5807	2.5916	2.5969	2.5465	2.5270	2.5398	2.5068	2.5254	2.5409	2.5929	2.7359	2.7335	2.7832	2.7459	2.7199	2.7556	2.7299	2.7934	2.7052	2.7149
Al	1.4355	1.4292	1.4193	1.4668	1.4922	1.4733	1.5059	1.4917	1.4764	1.4273	1.2504	1.2556	1.2099	1.2434	1.2736	1.2362	1.2599	1.1987	1.2789	1.2759
Sum(T)	4.0161	4.0208	4.0162	4.0133	4.0192	4.0131	4.0126	4.0170	4.0174	4.0202	3.9863	3.9890	3.9931	3.9893	3.9935	3.9918	3.9898	3.9921	3.9841	3.9908
Fe	0.0028	0.0028	0.0010	0.0024	0.0008	0.0034	0.0045	0.0029	0.0024	0.0024	0.0070	0.0031	0.0058	0.0054	0.0048	0.0057	0.0074	0.0073	0.0095	0.0141
Ca	0.3859	0.3828	0.3859	0.4253	0.4566	0.4356	0.4633	0.4472	0.4325	0.3738	0.2681	0.2833	0.2319	0.2592	0.2849	0.2573	0.2860	0.2176	0.3036	0.2947
Ba	0.0000	0.0000	0.0000	0.0000	0.0001	0.0000	0.0000	0.0001	0.0002	0.0000	0.0001	0.0005	0.0002	0.0003	0.0003	0.0001	0.0000	0.0002	0.0002	0.0002
Na	0.5909	0.5724	0.5776	0.5548	0.4977	0.5399	0.5177	0.5202	0.5344	0.5911	0.7507	0.7199	0.7539	0.7509	0.7152	0.7363	0.7090	0.7711	0.7114	0.6912
K	0.0028	0.0022	0.0032	0.0033	0.0026	0.0031	0.0020	0.0031	0.0027	0.0030	0.0043	0.0068	0.0082	0.0062	0.0050	0.0068	0.0051	0.0095	0.0050	0.0039
Sum	0.9823	0.9603	0.9677	0.9858	0.9580	0.9820	0.9875	0.9735	0.9722	0.9702	1.0302	1.0135	0.9999	1.0220	1.0103	1.0061	1.0074	1.0057	1.0297	1.0041
Or	0.29	0.23	0.33	0.34	0.27	0.32	0.21	0.32	0.28	0.31	0.42	0.67	0.82	0.61	0.50	0.68	0.51	0.96	0.49	0.40
Ab	60.32	59.78	59.75	56.42	52.01	55.17	52.67	53.60	55.11	61.07	73.38	71.28	75.85	73.89	71.16	73.60	70.90	77.25	69.75	69.83
An	39.39	39.98	39.91	43.25	47.72	44.51	47.13	46.08	44.61	38.62	26.21	28.05	23.33	25.50	28.34	25.72	28.60	21.80	29.76	29.78

Extended Data Table S5: Representative EPMA results of albite (wt%)

Sample Label	TS2										TS3									
											Oligoclase to albite					Albite				
No.	1	2	3	4	5	6	7	8	9	10	1	2	3	4	5	6	7	8	9	10
SiO2	64.07	64.13	63.59	63.85	64.16	63.90	64.28	63.84	64.28	64.24	62.74	63.00	64.13	63.40	64.04	63.43	64.28	65.30	65.21	65.25
Al2O3	21.86	21.69	21.66	21.77	21.94	22.00	21.49	21.98	22.19	21.59	22.18	21.69	21.48	22.05	21.69	21.94	21.19	20.64	20.71	20.79
FeO	0.17	0.19	0.15	0.19	0.14	0.14	0.19	0.21	0.25	0.25	0.70	0.45	0.22	0.10	0.16	0.31	0.16	0.15	0.21	0.24
CaO	2.47	2.45	2.32	2.56	2.53	2.54	2.23	2.48	2.49	1.97	4.94	3.63	2.32	2.68	2.24	3.10	1.95	1.08	1.27	1.21
BaO	0.01	0.00	0.01	0.00	0.01	0.01	0.00	0.01	0.00	0.01	0.01	0.00	0.00	0.01	0.01	0.01	0.01	0.01	0.00	0.01
Na2O	10.78	10.80	10.82	10.71	10.94	10.76	10.60	10.93	10.12	10.87	9.63	10.62	10.07	10.62	10.83	10.71	10.98	10.59	10.50	10.57
K2O	0.06	0.04	0.04	0.06	0.06	0.06	0.06	0.06	0.07	0.07	0.03	0.03	0.04	0.05	0.03	0.02	0.04	0.02	0.09	0.04
sum	99.41	99.29	98.60	99.14	99.77	99.41	98.85	99.50	99.39	98.99	100.23	99.42	98.25	98.91	98.99	99.52	98.62	97.79	98.00	98.10
	Structural formula on the basis of 8 oxygens																			
Si	2.8467	2.8521	2.8481	2.8455	2.8427	2.8400	2.8661	2.8375	2.8486	2.8622	2.7895	2.8168	2.8712	2.8324	2.8546	2.8250	2.8739	2.9242	2.9175	2.9159
Al	1.1446	1.1369	1.1436	1.1435	1.1454	1.1521	1.1294	1.1511	1.1591	1.1336	1.1623	1.1429	1.1335	1.1610	1.1394	1.1517	1.1168	1.0893	1.0921	1.0947
Sum(T)	3.9913	3.9890	3.9917	3.9890	3.9880	3.9921	3.9955	3.9886	4.0077	3.9959	3.9519	3.9597	4.0046	3.9933	3.9940	3.9767	3.9907	4.0135	4.0096	4.0107
Fe	0.0061	0.0069	0.0057	0.0070	0.0050	0.0051	0.0070	0.0077	0.0092	0.0093	0.0261	0.0169	0.0082	0.0039	0.0061	0.0116	0.0061	0.0055	0.0077	0.0088
Ca	0.1174	0.1168	0.1113	0.1224	0.1201	0.1211	0.1066	0.1181	0.1184	0.0941	0.2352	0.1738	0.1112	0.1284	0.1067	0.1478	0.0936	0.0517	0.0611	0.0579
Ba	0.0002	0.0000	0.0002	0.0000	0.0002	0.0002	0.0000	0.0002	0.0000	0.0002	0.0002	0.0000	0.0000	0.0002	0.0002	0.0002	0.0002	0.0002	0.0000	0.0002
Na	0.9287	0.9310	0.9399	0.9252	0.9395	0.9273	0.9167	0.9417	0.8694	0.9387	0.8303	0.9208	0.8737	0.9197	0.9358	0.9247	0.9522	0.9192	0.9109	0.9158
K	0.0034	0.0025	0.0025	0.0033	0.0031	0.0036	0.0033	0.0034	0.0038	0.0041	0.0016	0.0018	0.0022	0.0030	0.0015	0.0012	0.0021	0.0013	0.0054	0.0024
Sum	1.0558	1.0572	1.0596	1.0579	1.0679	1.0573	1.0337	1.0710	1.0008	1.0465	1.0934	1.1133	0.9954	1.0551	1.0504	1.0855	1.0541	0.9778	0.9850	0.9852
Or	0.32	0.24	0.23	0.31	0.29	0.34	0.32	0.32	0.39	0.40	0.15	0.17	0.23	0.28	0.14	0.11	0.20	0.14	0.55	0.25
Ab	88.49	88.64	89.20	88.04	88.41	88.14	89.29	88.58	87.67	90.53	77.81	83.98	88.51	87.50	89.63	86.12	90.87	94.55	93.20	93.82
An	11.19	11.13	10.56	11.65	11.30	11.52	10.39	11.10	11.94	9.07	22.04	15.85	11.27	12.21	10.22	13.77	8.93	5.32	6.25	5.94

Extended Data Table S6: Representative EPMA results of scapolite (wt%)

Sample La **TS2**											
No.	1	2	3	4	5	6	7	8	9	10	Average
SiO2	41.169	40.481	40.695	40.164	40.348	42.069	41.151	39.749	39.459	39.381	40.47
Al2O3	30.591	30.65	31.117	30.733	29.998	29.599	29.977	30.532	30.448	30.271	30.39
CaO	21.802	21.788	22.609	22.121	22.057	20.254	20.802	21.943	21.832	21.988	21.72
Na2O	0.714	0.673	0.524	0.354	0.331	1.198	0.752	0.293	0.259	0.229	0.53
K2O	0.00	0.00	0.00	0.00	0.00	0.00	0.00	0.00	0.00	0.00	0.00
Cl	0.01	0.01	0.01	0.02	0.00	0.01	0.01	0.09	0.01	0.01	0.02
sum	94.29	93.60	94.95	93.39	92.74	93.13	92.69	92.61	92.01	91.87	93.13
-O=Cl	0.00	0.00	0.00	0.00	0.00	0.00	0.00	0.02	0.00	0.00	0.00
Total	94.28	93.60	94.95	93.39	92.74	93.13	92.69	92.59	92.00	91.87	93.12
	Structural formula on the basis of $O_{24}Cl_1$										
Si	6.41	6.36	6.31	6.33	6.40	6.60	6.50	6.32	6.31	6.31	6.3840
Al	5.62	5.67	5.69	5.71	5.61	5.47	5.58	5.72	5.74	5.72	5.6512
Sum	12.03	12.03	12.00	12.03	12.00	12.07	12.08	12.03	12.04	12.02	12.0352
Ca	3.64	3.67	3.76	3.73	3.75	3.40	3.52	3.74	3.74	3.77	3.6718
Na	0.22	0.20	0.16	0.11	0.10	0.36	0.23	0.09	0.08	0.07	0.1624
Sum	3.85	3.87	3.92	3.84	3.85	3.77	3.75	3.83	3.82	3.85	3.8342
Cl	0.0053	0.0027	0.0047	0.0101	0.0011	0.0053	0.0037	0.0501	0.0043	0.0027	0.0090

Extended Data Table S7: Representative EPMA results of calcite (wt%)

Sample La **TS1**											
No.	1	2	3	4	5	6	7	8	9	10	Average
FeO	0.39	0.43	0.40	0.92	0.57	0.74	0.50	0.35	0.65	0.40	0.53
MnO	0.10	0.13	0.13	0.32	0.10	0.28	0.15	0.11	0.14	0.18	0.16
MgO	0.01	0.04	0.03	0.08	0.00	0.04	0.03	0.00	0.04	0.03	0.03
CaO	55.43	54.39	55.59	55.27	55.53	55.86	54.29	54.58	58.29	59.27	55.85
sum	55.93	54.98	56.15	56.59	56.20	56.93	54.97	55.04	59.12	59.87	56.58
Fe	0.0055	0.0060	0.0056	0.0127	0.0079	0.0102	0.0072	0.0049	0.0086	0.0052	0.0074
Mn	0.0014	0.0019	0.0019	0.0045	0.0014	0.0039	0.0021	0.0016	0.0018	0.0024	0.0023
Mg	0.0003	0.0009	0.0007	0.0019	0.0000	0.0011	0.0008	0.0001	0.0010	0.0007	0.0008
Ca	0.9929	0.9911	0.9918	0.9808	0.9907	0.9848	0.9899	0.9934	0.9886	0.9918	0.9896
Sum	1.0000	1.0000	1.0000	1.0000	1.0000	1.0000	1.0000	1.0000	1.0000	1.0000	1.0000

Extended Data Table S8: Representative EPMA results of omphacite (wt%)

Sample Label	TS3																
Comment		Diopside				Diopside to omphacite			Omphacite	Omphacite	Omphacite	Omphacite	Omphacite	Omphacite	Omphacite	Omphacite	Omphacite
No	1	2	3	4	5	6	7	8	9	10	11	12	13	14	15	16	17
SiO2	51.83	51.74	52.59	51.59	52.70	52.88	54.50	53.91	54.83	54.92	55.19	54.97	55.44	54.79	55.43	55.24	55.37
TiO2	0.18	0.12	0.14	0.14	0.09	0.12	0.12	0.10	0.13	0.11	0.10	0.12	0.15	0.15	0.16	0.17	0.14
Al2O3	4.84	5.47	6.36	6.79	6.47	6.86	6.93	6.95	7.33	6.69	6.85	7.37	7.21	7.33	7.38	7.25	7.35
Cr2O3	0.06	0.05	0.01	0.04	0.04	0.03	0.01	0.04	0.02	0.01	0.04	0.03	0.02	0.01	0.00	0.03	0.03
FeO	11.49	11.36	11.44	11.73	12.20	10.51	10.37	10.45	9.54	8.70	9.15	8.61	8.92	8.59	8.87	8.76	8.82
MnO	0.28	0.24	0.22	0.18	0.15	0.10	0.11	0.08	0.04	0.05	0.05	0.01	0.01	0.01	0.05	0.00	0.03
MgO	10.18	10.35	8.41	9.26	9.11	9.31	7.84	8.73	8.11	8.66	8.51	8.02	8.28	8.14	8.11	8.26	8.12
CaO	20.51	20.23	18.90	18.51	17.36	17.65	17.16	16.78	16.14	16.37	16.65	16.40	16.05	16.08	16.25	16.30	16.32
Na2O	1.35	1.36	2.16	2.23	2.65	2.86	3.61	3.72	4.46	4.57	4.66	5.03	5.10	5.13	5.22	5.26	5.40
K2O	0.00	0.00	0.00	0.00	0.00	0.00	0.00	0.00	0.00	0.00	0.00	0.00	0.00	0.00	0.00	0.00	0.00
sum	100.72	100.92	100.22	100.46	100.77	100.32	100.64	100.77	100.60	100.07	101.19	100.56	101.19	100.22	101.45	101.28	101.56
	Structural formula on the basis of 6 oxygens																
Si	1.9331	1.9221	1.9565	1.9203	1.9506	1.9520	1.9966	1.9756	1.9992	2.0090	2.0019	2.0013	2.0059	2.0008	2.0016	1.9991	1.9990
Al(T)	0.0669	0.0779	0.0435	0.0797	0.0494	0.0480	0.0034	0.0244	0.0008	0.0000	0.0000	0.0000	0.0000	0.0000	0.0000	0.0009	0.0010
Sum(T)	2.0000	2.0000	2.0000	2.0000	2.0000	2.0000	2.0000	2.0000	2.0000	2.0090	2.0019	2.0013	2.0059	2.0008	2.0016	2.0000	2.0000
Al(O)	0.1459	0.1614	0.2355	0.2183	0.2329	0.2504	0.2958	0.2759	0.3142	0.2883	0.2928	0.3161	0.3075	0.3155	0.3140	0.3083	0.3116
Ti	0.0049	0.0035	0.0039	0.0040	0.0024	0.0034	0.0034	0.0028	0.0036	0.0029	0.0028	0.0033	0.0041	0.0040	0.0043	0.0047	0.0038
Cr(3+)	0.0046	0.0039	0.0006	0.0028	0.0030	0.0026	0.0006	0.0030	0.0015	0.0010	0.0032	0.0024	0.0019	0.0011	0.0000	0.0026	0.0021
Fe	0.3585	0.3528	0.3558	0.3651	0.3778	0.3244	0.3177	0.3203	0.2910	0.2662	0.2774	0.2622	0.2699	0.2622	0.2678	0.2650	0.2662
Mn	0.0089	0.0075	0.0070	0.0055	0.0047	0.0030	0.0033	0.0025	0.0012	0.0016	0.0014	0.0004	0.0002	0.0002	0.0014	0.0000	0.0010
Mg	0.5658	0.5733	0.4667	0.5138	0.5027	0.5121	0.4281	0.4771	0.4406	0.4720	0.4600	0.4355	0.4465	0.4431	0.4364	0.4455	0.4369
Ca	0.8198	0.8053	0.7533	0.7380	0.6886	0.6982	0.6735	0.6589	0.6304	0.6414	0.6469	0.6396	0.6223	0.6293	0.6289	0.6320	0.6314
Na	0.0979	0.0980	0.1554	0.1608	0.1901	0.2044	0.2563	0.2646	0.3155	0.3238	0.3279	0.3554	0.3574	0.3634	0.3654	0.3692	0.3777
K	0.0000	0.0000	0.0000	0.0000	0.0000	0.0000	0.0000	0.0000	0.0000	0.0000	0.0000	0.0000	0.0000	0.0000	0.0000	0.0000	0.0000
Sum	2.0065	2.0055	1.9781	2.0083	2.0023	1.9987	1.9788	2.0051	1.9981	1.9973	2.0125	2.0148	2.0099	2.0188	2.0182	2.0274	2.0307
